# “Invisible” Conformers of an Antifungal Disulfide Protein Revealed by Constrained Cold and Heat Unfolding, CEST-NMR Experiments, and Molecular Dynamics Calculations

**DOI:** 10.1002/chem.201404879

**Published:** 2015-02-12

**Authors:** Ádám Fizil, Zoltán Gáspári, Terézia Barna, Florentine Marx, Gyula Batta

**Affiliations:** [a] Department of Organic Chemistry, Faculty of Science and Technology, University of DebrecenEgyetem tér 1, 4032 Debrecen (Hungary); [b] Pázmány Péter Catholic University, Faculty of Information TechnologyPráter u. 50A, 1083 Budapest (Hungary); [c] Department of Genetics and Applied Microbiology, Faculty of Science and Technology, University of DebrecenEgyetem tér 1, 4032 Debrecen (Hungary); [d] Medical University of Innsbruck, Biocenter, Division of Molecular BiologyInnrain 80–82, 6020 Innsbruck (Austria)

**Keywords:** conformation analysis, molecular modeling, NMR spectroscopy, protein folding, protein structures

## Abstract

Transition between conformational states in proteins is being recognized as a possible key factor of function. In support of this, hidden dynamic NMR structures were detected in several cases up to populations of a few percent. Here, we show by two- and three-state analysis of thermal unfolding, that the population of hidden states may weight 20–40 % at 298 K in a disulfide-rich protein. In addition, sensitive ^15^N-CEST NMR experiments identified a low populated (0.15 %) state that was in slow exchange with the folded PAF protein. Remarkably, other techniques failed to identify the rest of the NMR “dark matter”. Comparison of the temperature dependence of chemical shifts from experiments and molecular dynamics calculations suggests that hidden conformers of PAF differ in the loop and terminal regions and are most similar in the evolutionary conserved core. Our observations point to the existence of a complex conformational landscape with multiple conformational states in dynamic equilibrium, with diverse exchange rates presumably responsible for the completely hidden nature of a considerable fraction.

## Introduction

The mode of action of globular proteins is conventionally explained by their “functional” native structure. However, internal dynamics at various timescales has emerged as a key additional determinant of molecular function, ranging from fluctuations about a well-defined state to substantial conformational freedom exemplified by intrinsically disordered proteins (IDPs).[1–8] Moreover, recent NMR studies revealed low-populated, short-lived “excited” protein states[9–15] besides the dominant native structure, acting as active conformations in molecular recognition processes. For example, in a conformer selection-based partner recognition mechanism,[16–18] the native and thermodynamically more stable conformer may serve as a pool for supplying the required amount of low-populated, active conformers. Recent, extended-time (1 ms) molecular dynamics simulation of the small disulfide protein BPTI disclosed fluctuations between five conformational basins.[19] Studies of temperature-induced[20–23] or pressure-induced[24] protein unfolding support the existence of multiple states in a Trp-cage mini-protein,[25] a CylR2 homodimer,[26] and ubiquitin.[27]

In aqueous solution, many folded proteins exist in a reversible thermal equilibrium between folded and unfolded or partially folded states. The fraction of the folded conformation is generally the highest around room temperature and is highly dependent on the physicochemical environment, for example, temperature,[28] pressure,[24,29–31] pH,[32] and the presence of denaturing agents.[33,34] Detection and characterization of the unfolded fractions on the atomic level is difficult and requires indirect NMR techniques such as relaxation dispersion[35] and/or saturation transfer.[36–38]

Loss of globular structure in proteins at low temperatures (“cold unfolding”) has been demonstrated in numerous cases. It is generally accepted that cold unfolding of proteins is driven by the unique physicochemical properties of water, allowing parallel decrease of the entropy and enthalpy of a protein accompanied by the reorganization of its hydration shell at low temperatures.[28] Cold unfolding may be difficult to reach experimentally, because the low temperature melting point of the protein may be well below the freezing temperature of water. For one of the best characterized systems in this regard, the Yfh1 protein, three different states exist,[39] and the cold and high-temperature unfolded states have been shown to be similar but not identical.

Antimicrobial peptides populate the borderline[40] between the globular and disordered (IDP) protein world, which provides the motivation to study their unfolding. Antifungal disulfide proteins produced by filamentous ascomycetes are potential drug candidates for example, against aspergillosis.[41–45] These 50–60 residue proteins exhibit a beta-sheet dominated fold and are stabilized by three or four disulfide bonds. Although they are all abundant in basic residues, they differ in their specificity and mode of action.[46,47] However, the latter is not yet well understood at a molecular level. NMR solution structures of two representatives (PAF[48] and AFP[49]) have been determined so far, although neither have an explicit disulfide bond pattern. Recently, chemically synthesized[50] PAF could be used to unambiguously assign its previously elusive disulfide pattern. Given that PAF exhibits unusual stability over a year-long timespan,[48] it is an excellent novel model for stress-induced unfolding studies.

In this paper, we first present a more accurate solution structure of PAF, based on a nearly complete ^1^H/^13^C/^15^N NMR signal assignment and in accordance with its recently determined disulfide pattern. In addition, we performed a detailed NMR study on the hot and cold unfolding of PAF and performed experiments to identify hidden conformations that are in slow to intermediate exchange (millisecond timescale) with the major observable form. Our results reveal that, although this highly stable and disulfide-constrained protein remains largely structured under all conditions we applied, a number of different conformers are still likely to be present in dynamic equilibrium with each other. Importantly, the thermodynamics of unfolding differs significantly between some nonconserved loop and conserved core regions in PAF.

## Results and Discussion

### Solution structure of PAF with explicit disulfide bonds (Figure S1, in the Supporting Information)

In the knowledge of the unambiguous assignment of the disulfide pattern[50] and uniform ^13^C-^15^N labeling, more structural constrains were considered for the new structure determination. The refined structure does not differ significantly from the previous (2kcn) structure[48] (new code: 2mhv, RCSB:10362) with respect to its backbone conformation and secondary structure. However, disulfide bonds are given explicitly now, which lends further support to the ‘abcabc’ 7-36, 14-43, 28-54 pattern, and the backbone RMSD has decreased from 0.65±0.18 to 0.40±0.08 for the 20 conformer ensemble. The improvement is attributed to the explicit disulfide bonds and inclusion of dihedral angle constraints using Cα, Cβ ^13^C chemical shifts and TALOS+.[51] The RMSD difference between the unconstrained (2kcn) and disulfide constrained (2 mhv) ensembles was 1.31±0.58, and mainly arises from differences in loop 3, between cysteines 28 and 36. By using the new structure and previously obtained ^15^N order parameters,[48] we calculated a structural ensemble reflecting the conformational heterogeneity of PAF at the pico- to nanosecond timescale.

### PAF does not undergo complete unfolding

**Figure 1:**
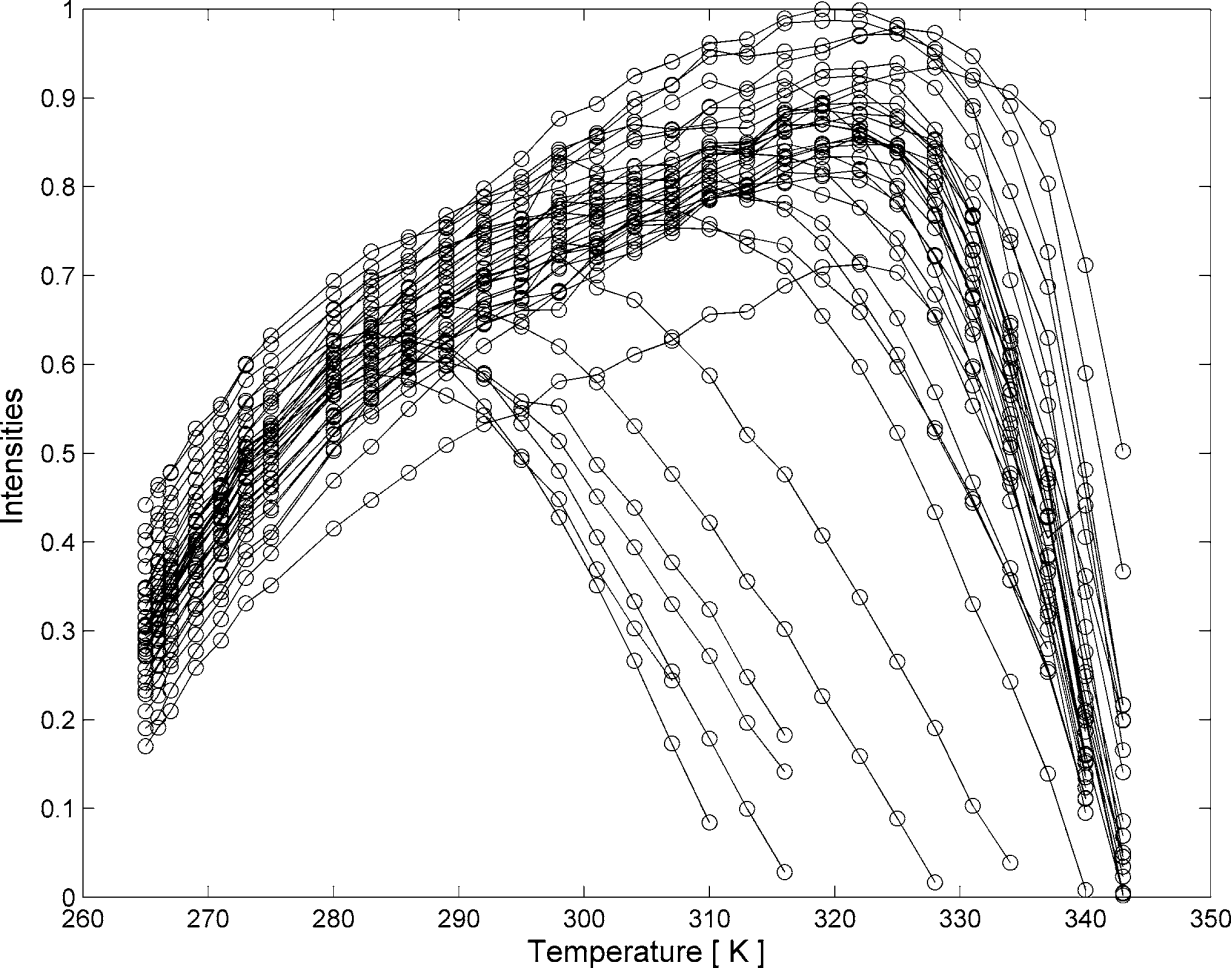
Raw ^15^NH-HSQC signal integrals of PAF as a function of temperature, normalized to the most intense peak. Volume integrals were corrected against temperature and ^1^H-pulse calibration according to ‘PULCON’ protocol.[52–54]

From the 50–800 ppb drift of the combined NH chemical shifts ΔNH=((ΔH)^2^+(ΔN/6.5)^2^)^1/2^ upon temperature changes, it is clear that the PAF conformer that is visible by NMR spectroscopy largely preserves its secondary and tertiary structure during thermal stress, and that the overall 3D structure changes continuously (conformational drift model). Our observations show that covalent disulfide bonds prevent complete (irreversible) unfolding of PAF in a temperature range from 258 to 347 K (the lowest temperatures were reached by supercooling in 1 mm capillaries). Both the ^15^N-^1^H and ^13^C-^1^H HSQC spectra indicated the presence of substantial numbers of structured regions at all temperatures investigated, except at the very ends of the temperature range. At the cold end, neither the collapse of the ^15^N-HSQC spectrum, nor new peaks were observed due to the constrained nature of unfolding. When we use the integral intensity of the largest peak (K42) as reference 100 % at pH 6 (considering all ^15^N-^1^H HSQC spectra in the entire temperature range), then the highest peak intensities for all residues gives an average of 86±7 % (Figure 1). However, pH also influences the peak volumes due to the different exchange rates of the amide protons. pH titration of PAF revealed that the global maximum for the intensities of all NH peaks is approximately pH 6.0, except for a few residues (K2, S10, K11, D19, D23, D32, N33, Y48) that are close to maximum intensity at approximately pH 4. Throughout all the experiments the observed temperature-induced changes proved to be reversible.

### The observed cold and hot structures are different from the native structure and from each other

The chemical shifts observed for the hot (344 K) and cold (268 K) states indicated that the structures at the two temperature extremes investigated differ from each other. It is expected, however, that the ensembles of conformations in these states largely overlap, and the effects of temperature change, structural alterations, and population drifts are all represented in the observed chemical shift changes. To investigate this in more detail, we designed an ensemble selection approach based on a computer generated structure pool based on chemical shifts. By repeating the ensemble selection process 10 000 times, we investigated the conformers with the largest difference in their selection rate for the hot and cold states (see Experimental Section). These structures, although not precise models, are expected to represent the conformers for which differential distribution at the two temperatures is mainly responsible for the observed differences between the hot and cold unfolded ensembles present. Our analysis reveals that the primary source of structural heterogeneity between the states can be found at the loop and terminal regions.

We found from the thermal unfolding experiments that the nonconserved loop regions are the most sensitive against heat shock, whereas cold unfolding occurs concertedly at all residues (Figure 1 and Figure S51 in the Supporting Information).

### Analysis of unfolding and quantitative NMR indicates that NMR-invisible conformers can constitute up to 30 % of conformer population

The cold and hot unfolding of PAF has been monitored over a broad temperature range (265–344 K) by using ^15^N and ^13^C spy nuclei for each resolved site. The population of the folded conformer is reflected in the observed HSQC NMR peak volumes, and they exhibit maxima at intermediate temperatures (highest stability temperature). Given that the average *T*_2_ relaxation times are in the 100–300 ms region (see the Supporting Information, Table S6), and the ^1^*J*_NH_ spin-spin coupling is rather homogeneous over the sequence, and the sum of INEPT periods is approximately 22 ms, the signal loss of the transversal magnetization is not significant, and must be uniform for most residues. For the same reason, different line broadenings of *T*_2_ origin cannot influence peak volumes under the present integration routines (see the Supporting Information, Figure S53 a,b). Apparent differences between peak intensities arise mostly from pH effects, as discussed above. However, the pH is fixed during temperature changes, therefore maximum intensities can be conveniently normalized to identical values. It is estimated that the temperature dependent pH change in the 265–344 K range is below 0.25 with our buffer,[55–58] which could result in less than 1 % change of the sum of all peak volumes and can therefore be neglected. Changing amide exchange rates can also influence NH peak volumes, which was minimized by keeping the water magnetization in the +*z* direction before acquisition in the HSQC experiment. Although it is difficult to assess the effects of exchange with the solvent at low and high temperature, careful experimental design is sufficient to monitor the unfolding events. Minor conformers may remain NMR invisible because they are either low populated[59] or they have a chance to be in the intermediate exchange regime (either on the ^1^H, or the ^15^N chemical shift timescale), thereby giving small, broad signals that are hidden in the baseline (see the Supporting Information, Figure S50). In the case of PAF, we estimate from the error minimization of unfolding experiments, that invisible conformers may populate as much as 30–40 % compared with the visible conformers, even at the highest protein stability temperature (300 K). The amount of protein in the invisible “thermal” conformations was reported to be 8 % for a heat shock protein[23] and 37 % for yeast frataxin.[22]

### Heat and cold unfolding of PAF is site-specific

**Figure 2:**
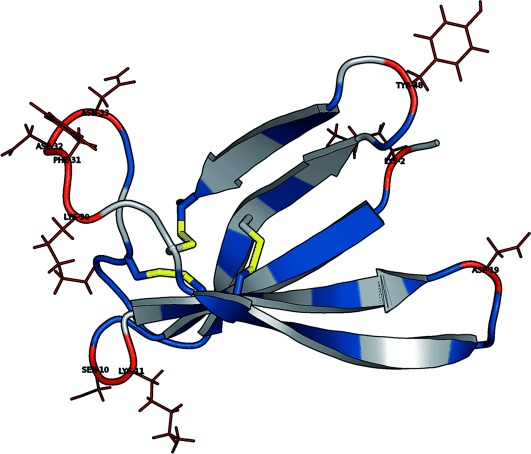
Top) Ribbon representation of PAF, displaying the error limits of the thermal unfolding experiment fits. Red: two-state model works, blue: three-state model also works; gray: not detected residues. Bottom) Sequence alignment of representative PAF homologs. Highlighted residues show satisfactory agreement with the two-state model in PAF and correspond to nonconserved loop sites in homologs.

**Figure 3:**
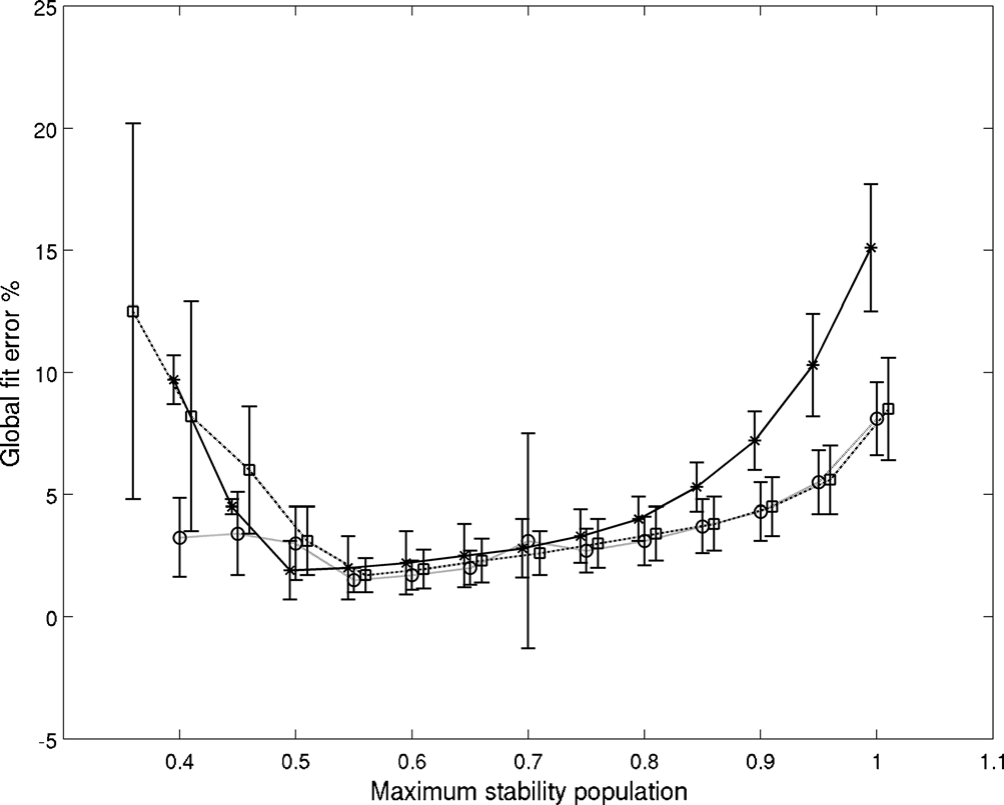
Global fitting errors as a function of maximum stability populations from ^15^N-HSQC spectra. Models are labeled as “FU”=*, “FIU”=□, “FU_2_”=○. Data points are shifted slightly for clarity.

**Figure 4:**
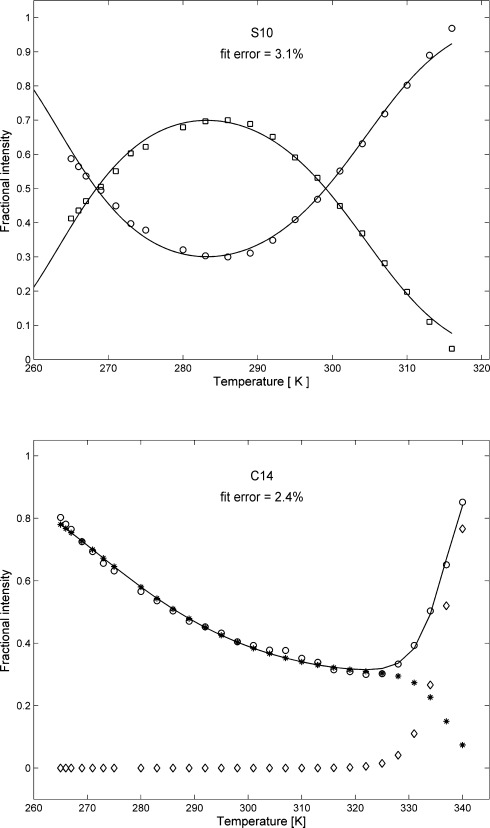
Fitting of the HSQC peak integrals as a function of temperature according to pertinent thermodynamic models. Top) Fitting of Ser 10 with the two-state model: both folded (□) and unfolded (○) fractions are shown. Bottom) Fitting of Cys 14 with the three-state model [the intermediate u1 (*) and the unfolded u2 (◊) populations are shown separately as well as their sum u1+u2 (○)].

Analysis of the integrated intensities of HSQC peaks[60] at different temperatures yielded a somewhat unexpected result: Signal intensities decrease at temperatures away from approximately 300 K, but the range in which maximal or near-maximal intensity can be observed is not uniform for all residues. In the ^15^N-HSQC spectra, the temperature range for which maximum peak volumes are observed is much wider for conserved core residues than for some of those in the nonconserved loop regions (Figure 2; we refer to residues K2, S10, K11, D19, K30, F31, D32, N33, Y48 as group 1, whereas group 2 means all other observed residues). This is in line with the observed low-temperature melting points (*T*_low1_=271±4 K and T_low2_=281±4 K) and maximum stability temperatures (*T*_max1_=289±7 K) for group 1 residues and (*T*_max2_=306±4 K) for group 2 (see the Supporting Information, Table 1–3). Moreover, only group 1 residues could be adequately fitted (below a 6 % error limit) with the accepted two-state Becktel–Schellman thermodynamic model[61] as a function of temperature [see the Supporting Information, Figure S4–12, Eq. (1–4)]. The two-state model gives extreme thermodynamic parameters at group 1 residues (see the Supporting Information, Figure S13–18). The application of three-state models significantly improved the quality of the fits for all residues (<5 %) including those in the nonconserved loop regions (<2.5 %; see below, Figure 4)). By using a three-state model (FIU) [see the Supporting Information, Eq. (5–16)] with an intermediate **I** state, we double the number of thermodynamic parameters that could be interpreted and compared to the simple two-state analysis. The three-state model predicts energetically similar F–I transitions for group 1 residues as the two-state model, because enthalpies and heat capacities are in the same range (Δ*H*_u1_=65±13 vs. 66±9 kJ M^−1^, and Δ*C*_p_=1.8±1.1 vs. 3.7±1.1 kJ M^−1^ K^−1^). The agreement between the two models is still reasonable for group 2 (see the Supporting Information, Table 1–3). However, for the second, I-U unfolding, the values of Δ*H*_u2_ are much higher and more scattered for both groups, but with more pronounced differences between the two events in group 2 residues, suggesting that the I-U transition is more hindered in residues of group 2. Thus, the I-U events may be tentatively related to those states preceding the breaking of the disulfide bonds. It should be noted that the same data can also be fit without an intermediate state using a putative FU_2_ model with two independent unfolded states, u1 and u2. Complementary NMR unfolding experiments with ^13^CαHα spy nuclei did show the unfolding effect, however the thermodynamic parameters obtained were different from ^15^N—H observations and there was no appreciable difference between group 1 and group 2 residues (see the Supporting Information, Table S2 and Figure S20–30). Interestingly, in our case, complementary electronic circular dichroism (ECD) spectra report only on the hot unfolding as a function of temperature (see the Supporting Information, Figure S52 a and S52 b). This is because the ECD spectra are dominated by the dihedral angles of the highly stable disulfide bonds buried in the hydrophobic core, and not by the beta sheets of PAF. However, in other cases, ECD and other techniques[62] provided invaluable support for the NMR observations. Global error analysis of different models showed that, independent of model selection, the minimum fit error is achieved when the maximum population of the visible conformer is around 70 % (Figure 3). The amount of the hidden states in PAF was independently estimated by quantitative NMR spectroscopic analysis by using an unstructured ^15^N-Ala labeled peptide model of a RAQI sequence, that has one ^15^N–^1^H HSQC peak at pH 3.4. By using known concentrations of the tetrapeptide and PAF, and measuring two separated CH_3_ groups at the lowest chemical shifts in each of their 1D ^1^H NMR spectra, we found only 2 % difference between the expected concentrations. In contrast, comparing the HSQC signal intensities, the PAF concentration was 20–27 % lower than that of the peptide, using arbitrarily chosen PAF peaks (C28/N40/K42/T47 or N40/T47).

### Model fits reveal sites with large chemical shift difference relative to the visible state

The fit of experimental chemical shifts as a function of temperature was first carried out by using the populations derived from the two and three-state model fits (Figure 4), with all conformers contributing to the observed chemical shifts proportional to their population (see the Supporting Information, Figure S31–40). This phenomenological approach is based on a conformational drift model, supposing linear chemical shift dependence of all conformers as a function of temperature. By using the two-state model and assuming that the observed chemical shifts originate from the weighted average of the folded (F) and—invisible—unfolded (U) conformers, the ^15^N “shifts” of the two states can be separated as a result of fits. The largest overall shift differences between U and F states were obtained for residues 3, 47, 54, and 55, close to the PAF termini. It is important to note that none of the observed chemical shifts (^15^N or combined ^15^NH) could be adequately fit with temperature-independent chemical shifts of the folded and unfolded states, in contrast to the generally accepted approach[25] used in case of fast exchange limit. Unfortunately, the “chemical shifts” obtained this way cannot be directly applied to structure determination.

### Sensitive CEST experiments are capable of detecting low-populated, slowly exchanging hidden conformers

**Figure 5:**
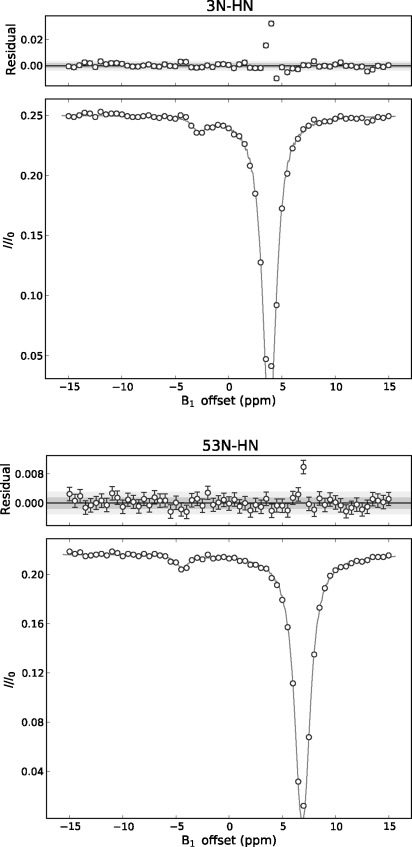
^15^N-CEST profile of terminal Asp 53 (bottom) and Tyr 3 (top) residues fitted with ChemEx code.[38] The small peaks represent low populated protein fractions at specific ^15^N chemical shifts in slow exchange with the visible native conformer.

We ran ^15^N-CEST experiments (CEST=chemical exchange saturation transfer)[36,38] at 298 K, and pH 6.0 and observed hidden exchange partners around both termini of PAF, in some cases with remarkable ^15^N chemical shift differences (Figure 5). The apparently affected residues are Tyr 3, Thr 47, Ala 51, Asp 53, and Cys 54 (see the Supporting Information, Figure S41–46). Although the CEST peaks are small, simultaneous fit[38] of the five residues with a two-state model yielded the following results: exchange rate *k*_ex_=165±62 s^−1^, population of the hidden conformer *p*_B_=0.15±0.02 %, and chemical shift offsets relative to the main peaks: Tyr 3=−6.7±0.3, Thr 47=−5.9±0.2, Ala 51=5.0±0.2, Asp 53=−11.3±0.4, and Cys 54=−4.4±0.3 ppm. It is remarkable that such a low population state could be observed and analyzed with reasonable accuracy. However, in spite of the significant changes in ^15^N shifts, in the absence of more data, their use for structure determination is limited.

Notably, three of the CEST-sensitive residues coincide with those that show the largest estimated shift differences between F and U states. Key features of the putative “excited state” models indicate rearrangement of the C-terminal beta-strand and disruption of spatial contacts between loop 4, containing Thr 47, and the N-terminus (see the Supporting Information, Figure S48). Considering the large ^15^N chemical shift difference at D53 with respect to the magnitude of the changes obtained from molecular dynamics, the question arises whether disulfide shuffling[63] or isomerization could be involved, as seen in BPTI.[35]

### The native state of PAF is a complex ensemble with diverse dynamics

**Figure 6:**
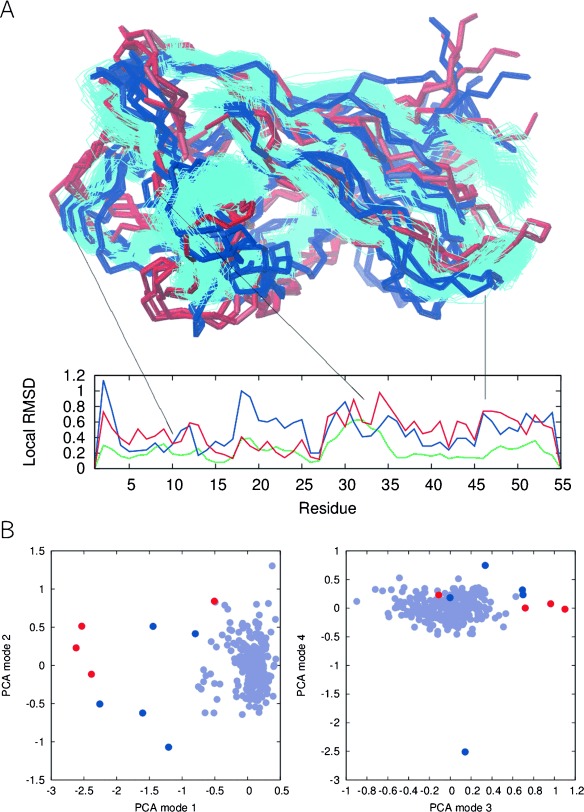
Top) Backbone representation of generated PAF conformers. Cyan: MUMO16 ensemble reflecting the pico- to nanosecond timescale dynamics of PAF. Red and blue: Conformers characteristic for the hot and cold states, respectively. Middle) Local RMSDs between average structures derived from the populations: observable vs. hot state-specific (red line), observable vs. cold state-specific (blue line), hot state-specific vs. cold state-specific (green) structures. Bottom) Principal component analysis of the conformers shown, colored accordingly.

Our results show that even at the temperature of highest stability, PAF can only be described as an ensemble of interconverting visible and invisible conformers (Figure 6 and Tables S4 and S5 in the Supporting Information). Our model fits indicate that the factors influencing the chemical shifts of PAF have linearly changing characteristics in a broad temperature range. These factors probably include local geometry, H-bonds, solvation effects, and exchange with low populated states. Thus, the native state of PAF might be described by the conformational drift model. Our attempts to identify slowly exchanging ‘invisible’ conformations revealed one such partner with exchange kinetics on the millisecond timescale. Thus, the emerging complex picture is that about 70 % of PAF gives rise to the NMR-visible ^15^NH signals and this corresponds to a well-folded (native) conformer with restricted dynamics and no detectable exchange on the pico- to nanosecond timescale (see the Supporting Information, Figures S2 and S3). However, a major fraction of the remaining approximately 30 % must be in intermediate exchange with this conformer (causing invisibility). In addition to this, a very low populated fraction is clearly found to be in slow exchange with the native PAF conformer. Only the less constrained terminal regions gave significant chemical shift effects in CEST experiments, therefore, we could not obtain detailed structural data on the low-populated hidden state and can only provide a highly approximate structural model based solely on moderately informative ^15^N chemical shifts (see the Supporting Information, Figure S47 and Table S5). Nevertheless, our observation that three residues (Tyr 3, Thr 47 and Cys 54) are in a chemical environment that differs from that in the visible state both in the “thermal” and in the CEST-detected exchange partner raises the possibility that these states are not completely independent and may even be on the same pathway.

### Implications for NMR data interpretation and submission practice

If significant amounts of dynamically diverse invisible conformers are generally present in the solution of globular proteins at maximum stability temperature—as in our case—then the measured NMR parameters are only representative of the particular “NMR-visible” conformer. It is also clear that by shifting the temperature or other physicochemical boundaries, chemical shifts or coupling constants may arise from even smaller fractions of the dissolved protein. This can potentially hinder the interpretation of NMR measurables derived from MD calculations and invalidate protein concentration measurements that are obtained with simple protocols.

### Hidden conformers and PAF function

Details of the mode of action of PAF remain elusive but it seems to be distinct[46,64] from its closest homologue AFP. PAF induces plasma membrane hyperpolarization in sensitive fungi. In the absence of identified interaction partner molecules, we do not yet have information about the bound conformation(s) or whether they fall within the variability of the pico- to nanosecond timescale dynamics of the observable conformer or are more similar to the thermally ‘unfolded’ states or perhaps to the low-populated “CEST-conformer”, or even whether all of these have a specific functional role (preliminary antifungal activity studies with PAF exhibited maximum activity around 298 K). One plausible scenario is that the visible state serves as a pool for supplying molecules that maintain the level of hidden conformers that are directly involved in functional interactions. In this case, understanding of PAF function at the molecular level requires consideration of its complex dynamics in the native state, including conventionally unobservable species.

## Conclusions

Studying cold and hot unfolding in disulfide proteins is particularly interesting because they do not show all the characteristics of cold unfolding. On the other hand, the simple two-state FU model works well only for a limited set of residues in PAF. Not surprisingly, these nonconserved residues lack strong H-bonding, and therefore they are the most exposed to solvent exchange and pH effects (see the Supporting Information, Figure S49). Extension to two possible three-state models results in significant improvement in the fits of all residues, but it is difficult to decide between the FIU and FU_2_ unfolding mechanisms. Independent of model selection, the fits are best when significant numbers of NMR invisible conformers are supposed at maximum stability temperature. Furthermore, independent of model selection, we found that conformational drift of ensembles is driven by temperature.[65] Unfortunately, the results of chemical shift fitting of our unfolding experiments cannot be directly applied for structure determination. In case of PAF, the conformational drift model seems to work for both visible and invisible states in a broad temperature range. Methodologically, one might argue that “invisible” conformers do not contribute to observable peak volumes, however they do contribute to some extent to the apparent chemical shifts. This can be explained by the fact that integration is limited to observable peaks (fading effect), whereas the chemical shifts are measured with high precision, and they sense contributions from exchange partners, the populations of which change by temperature (as shown by three-site exchange simulations; see the Supporting Information, Figure S50). Special care must be taken with the experimental and data processing conditions of ^15^N-HSQC experiments because local unfolding events and dynamics are very complex, and because the thermodynamic parameters may depend on the features of the actual detector.[62] However, combining stress-induced unfolding experiments with exchange-sensitive NMR techniques such as CEST or CPMG-RD and in silico molecular dynamics together is a promising way to map the diverse conformational dynamics of proteins in solution.

## Experimental Section

**Production of PAF**: For the preparation of ^13^C-/^15^N-labeled PAF, *P. chrysogenum* Q176 (ATCC 10002) was cultivated in minimal medium (MM: 0.3 % Na^15^NO_3_, 0.05 % KCl, 0.05 % MgSO_4_⋅7 H_2_O, 0.005 % FeSO_4_⋅7 H_2_O, 1 % ^13^C-d-glucose, 25 mm phosphate buffer, pH 5.8) at 25 °C on a rotary shaker at 250 rpm. Na^15^NO_3_ and ^13^C-d-glucose were purchased from Cambridge Isotope Laboratories Andover, Mass., USA.

PAF was isolated by molecular weight (MW) filtration and ion-exchange chromatography. In brief, the supernatant of a 72 h culture was cleared by centrifugation for 30 min at 10 000×g, 4 °C, ultrafiltered through a YM-30 membrane (Millipore, Bedford, Mass., USA) in an 8.200 Amicon stirring cell and loaded on a CM-sepharose CL-6B column (Amersham, Uppsala, Sweden), which had been equilibrated in 10 mm Na-phosphate buffer, 25 mm NaCl, 0.15 mM EDTA, pH 6.6. The protein was eluted by 200 mM NaCl. The PAF-containing fractions were pooled, dialyzed against water, concentrated in Centriprep YM-3 filter devices (Millipore), and filter-sterilized (Millex GV filters, 0.22 mm, Millipore) before lyophilization. NMR samples were prepared under the same conditions as previously reported.[48] In brief, 3 mg of lyophilized protein was dissolved in 270 μL buffer (10 mM Na_2_HPO_4_/NaH_2_PO_4_ pH 6.0 buffer containing 5 % D_2_O, 0.04 %NaN_3_ and 40 mM NaCl) and filled into a Shigemi NMR tube. Hence the final protein concentration was 1.75 mM, which was required to reach adequate sensitivity.

**NMR experiments**: Measurements for resonance assignment were performed with Bruker Avance II 500 and 700 MHz (the latter for the NOESY type experiments) spectrometers equipped with TXI z-gradient probeheads. All spectra were acquired at 298 K. Spectra were processed with Topspin 3.0 software. Sequential resonance assignment was performed with CARA 1.8.4 software.[66] By using 2D ^15^N-HSQC[67,68] as root, triple resonance 3D HNCO,[69] HN(CA)CO,[70] HNCA, HN(CO)CA,[69] HNCACB,[70] and HN(CO)CACB[71] spectra were used for identification of intra and inter-residual connections through the protein backbone. All backbone carbon resonances were assigned this way with the exception of Pro-29. 3D HNHA,[72] HBHA(CO)NH,[73] HNHA(CO)NH, HCC(CO)NH,[74] HC(C)H-COSY, (H)CCH-TOCSY, and HC(C)H-TOCSY[75] experiments were used for side-chain assignments. For aromatic proton assignment, 2D CB(CGCD)HD and CB(CHCDCE)HE[76] spectra were used. Side-chain carboxamide protons of asparagine residues were identified from the 2D-NOESY. Both 2D and 3D ^13^C and ^15^N edited NOESY spectra were collected using 130 ms mixing time. Completeness of backbone and side-chain assignment reached 96.7 and 72.7 %, respectively. Chemical shifts were referenced to external 4,4-dimethyl-4-silapentane-1-sulfonic acid (DSS) as reference compound.[77,78]

All proton assignments were finely adjusted to their peak maximum in the 3D ^13^C-resolved and ^15^N-resolved NOESY-HSQC spectra measured at 700 MHz to achieve automatic NOESY peak picking and assignment. ATNOS-CANDID algorithm was used in combination with Cyana 2.1 software for structure determination.[79,80] Setup files were prepared with the aid of the UNIO’10 platform. Disulfide pairing was fixed in the Cyana input according to the native C7-C36, C14-C43, C28-C53[50] disulfide pattern. Diastereotopic proton assignments for nonequivalent CH_2_ groups were not given (BMRB entry 19657). Backbone torsional angle constraints were considered according to TALOS+.[51]

**Temperature-dependent experiments**: For precise measurement of amide cross-peak volumes, sensitivity-improved ^15^N-HSQC[67,68] was used for a 1.75 mM ^15^N-PAF sample with 2.5 s delays between scans. For measuring Cα—Hα, Cβ—Hβ and some other side-chain C—H cross-peak volumes, constant-time ^13^C-HSQC experiments[81] were used for a ^15^N/^13^C-PAF sample. Every new dataset was acquired after suitable stabilization of the spectrometer at that temperature. The probehead was tuned and the pulses were calibrated at each temperature. The ^1^H 90° pulses were in the range of 10–15 μs, whereas the ^15^N pulse was fairly constant at 37 μs. Some control experiments were carried out with 5 s relaxation delay and the observed peak volume changes were below 5 %. All spectra were Fourier-transformed to 2048×1024 datapoints using cos^2^ window function in the ^1^H and ^15^N dimensions. The chemical shifts of the cross-peaks vary with the temperature; therefore, assignments had to be transferred. To this end the Topspin peak lists were exported and further processed by an in-house written MATLAB script. In the case of ^13^C CT-HSQC experiments, assignment transfer was done using the CCPN[82] copy function. Least-squares fitting was performed with MATLAB scripts. Fit errors are derived from the scatter of the difference between experimental and fitted data.

^**15**^**N-CEST experiments**: ^15^N-HSQC-T_1_-type experiments were run as described[38] using 61 selective ^15^N-irradiation frequencies (0.5 ppm resolution) in separate 2D experiments and one reference with 2 ms “saturation”, without irradiation. The ^15^N oscillating field strength of γB1=25 Hz was generally applied, and in some cases double or half of this. In separate experiments, four scans were applied and 128 increments in the nitrogen domain, interscan delay was 1.7 s whereas soft irradiation was of 0.4 s duration. 62 experiments lasted approximately one day, the experiments were run in triplicate and the data were co-added. The cross-peak volumes were evaluated by using Topspin 3.1 and transferred for further processing by matlab scripts. The experiments were simultaneously fit (Figure 5) for the selected five residues with the code ChemEx, kindly provided by Dr. G. Bouvignies.

**Structural ensembles**: Structural ensembles reflecting the pico- to nanosecond timescale dynamics of the visible PAF state were calculated by using the refined PAF structure, NOE and previously determined S2 data. S2 restraining and pairwise treatment of NOE distances over replicas similar to the MUMO protocol were implemented in GROMACS 4.5.5. The AMBER99SB force field[83] and TIP4P water model were used. Structures were extracted every 200 ps from a 4 ns simulation with 16 replicas. By discarding conformers before the first 1 ns of the run, 256 structures were selected for the final ensemble. Correspondence to experimental data was verified by using the CoNSEnsX web server.[84]

Approximate models for the states characteristic of the hot and cold states were obtained based on ^13^Cα and amide ^15^N chemical shifts by selecting conformers from a pre-generated pool. The accelerated molecular dynamics (AMD)[83] Scheme for dihedrals was implemented [see the Supporting Information, Eq. (17)] in GROMACS 4.5.5 to achieve an enhanced sampling of the conformational space, thus supposedly covering states occurring on slower timescale (micro- to millisecond) motions. A conformer pool with 2001 members including the starting model (corresponding to the native structure) was generated with dihedral boost energy of 5000 kJ mol^−1^ and an alpha value of 100, extracting conformations every 20 ps from a 40 ns run. For these calculations, the AMBER99SB force field was used with the GBSA implicit solvent model. For each conformer in the pool, chemical shifts were predicted with SHIFTX2.[85] In the next step, a random selection algorithm was used to select a sub-ensemble that corresponds to the observed chemical shifts of the given state. Instead of a fixed size for the final ensemble, only a minimal size of 2 for the target ensemble was used. After randomly selecting an initial set of conformers, eliminations and additions from the pool were performed (requiring that the agreement with experimental data increases at each step), and a maximum of 10 000 steps were allowed. For each state, two runs were performed as the agreement with experimental chemical shifts was monitored by calculating either simple Pearson correlation or Q-factor. This selection procedure was repeated 10 000 times, and the conformers most often selected in ensembles with the chosen measure (correlation or Q-factor) above the average for each of the hot and cold states were listed. Conformers with at least twofold difference in their occurrence between the two states (hot and cold) for both the correlation and Q-factor-selected ensembles selected as exclusively representative for the cold or hot state (GROMACS source code files containing all of the modifications used are available at http://users.itk.ppke.hu/gaszo.)
