# Supplementary material for: “Invisible” Conformers of an Antifungal Disulfide Protein Revealed by Constrained Cold and Heat Unfolding, CEST-NMR Experiments, and Molecular Dynamics Calculations
Source: Chemistry. 2015 Feb 12;21(13):5136–44. doi: 10.1002/chem.201404879 (PMC4464532; doi:10.1002/chem.201404879)
Supplement: Supplementary file 1 [file chem0021-5136-sd1.pdf]

CHEMISTRY
A European Journal

Supporting Information

"Invisible" Conformers of an Antifungal Disulfide Protein Revealed by Constrained Cold and Heat Unfolding, CEST-NMR Experiments, and Molecular Dynamics Calculations

Ádám Fizil,[a] Zoltán Gáspári,[b] Terézia Barna,[c] Florentine Marx,[d] and Gyula Batta*[a]

chem_201404879_sm_miscellaneous_information.pdf

I. Supplementary methods

a. *Description of the thermodynamic models*

The temperature dependent equilibrium between the folded and unfolded state of a protein (two-state model) was originally described by Becktel and Schellman[1], Privalov and Gill[2,3] and recalled later by Szyperski[4]. The basic assumption in these thermodynamic equations is that the heat capacity change at constant pressure (ΔC_p) is temperature independent, while changes of other thermodynamic quantities (enthalpy ΔH, Gibbs potential ΔG, and entropy ΔS) are not. Experimentally, the amount of unfolded protein fraction ($f_{u,T}$) is monitored as a function of temperature and the free enthalpy ($\Delta G_{u,T}$) of unfolding can be given with the Gibbs-Helmholtz equation:

$$\text{Eq. 1.} \quad \Delta G_{u,T} = \Delta H_{u,T_h}\left(1 - \frac{T}{T_h}\right) + \Delta C_p\left(T - T_h \ln\frac{T}{T_h}\right)$$

and the equilibrium constant between the “unfolded” (u) and folded (“native”, n) states is

$$\text{Eq. 2.} \quad K_{u,T} = \exp\left(\frac{-\Delta G_{u,T}}{RT}\right) = \frac{f_{u,T}}{f_{n,T}}$$

$$\text{Eq. 3.} \quad f_{u,T} + f_{n,T} = 1$$

and therefore

$$\text{Eq. 4.} \quad f_{u,T} = \frac{K_{u,T}}{1+K_{u,T}}$$

Using the nested equations of 1 and 2 the measured unfolded (or folded) protein fraction as a function of temperature can be fitted against the unknown thermodynamic parameters of two-state unfolding. $\Delta G_{u,T}$ is the difference between two big (enthalpic and entropic) terms, and has an inverted parabolic profile with a maximum at the highest folded protein fraction (max [$f_{n,T}$]) at the temperature of maximum stability T_{max}. In addition to the high temperature melting point T_h, there is a low temperature melting point T_l, that is often well below the freezing point of water. At melting temperatures $\Delta G_{u,T} = 0$ (zero-crossing) and populations are equal $f_{u,T} = f_{n,T} = 0.5$. Many reversible two-state folders have maximum stability around room temperature[5] $T_{max} = 293 \pm 8$K. However, other thermodynamic parameters (e.g. T_h and T_l) are quite diverse according to the different shapes of the parabolic $\Delta G_{u,T}$ curves. Though difficult to measure the exact amount of the “unfolded” fraction of a protein at the maximum stability temperature (T_{max}), it may be well around 8-37% [4,6]. If such observations are generally valid, it may impact or views on protein machineries.

In cases like the temperature induced unfolding of PAF the NMR fading effect (exchange peaks hidden in baseline) dominates the exchange and the available measurables cannot be easily utilized for disclosing all mechanistic details of unfolding. First we assume a thermal equilibrium between three-states as N↔U1↔U2. As a proof of concept we consider here the exchange between a folded (n) conformer and the “unfolded” NMR invisible conformers u_1 and u_2 via the u_1 intermediate state.

The equilibrium constants for the two exchanges are K_1 and K_2:

Eq. 5. $$K_1(T) = \frac{u_1(T)}{f_n(T)} \;,\; K_2(T) = \frac{u_2(T)}{f_{u1}(T)}$$

Eq. 6. $$f_n(T) + f_{u_1}(T) + f_{u_1}(T) = 1$$

then the total unobserved fraction is:

Eq. 7. $$u = f_u(T) = f_{u_1}(T) + f_{u_2}(T)$$

and the folded fraction is

Eq. 8. $$f_n(T) = 1 - u = \; [1 + K_1(T) + K_1(T)K_2(T)]^{-1}$$

Eq. 9. $$u = K_1(T)f_n(T) + K_1(T)f_n(T)K_2(T) = K_1(T)f_n(T)[1 + K_2(T)]$$

Eq. 10. $$u = K_1(T)(1-u)\big(1 + K_2(T)\big) = K_1(T) + K_1(T)K_2(T) - uK_1(T) - uK_1(T)K_2(T)$$

Eq. 11. $$u[1 + K_1(T) + K_1(T)K_2(T)] = K_1(T) + K_1(T)K_2(T)$$

Eq. 12. $$u = \frac{K_1(T)[1+K_2(T)]}{1+K_1(T)+K_1(T)K_2(T)}$$

Eq. 13. $$u1 = \frac{K_1(T)}{1+K_1(T)+K_1(T)K_2(T)}$$

Eq. 14. $$u2 = \frac{K_1(T)K_2(T)}{1+K_1(T)+K_1(T)K_2(T)}$$

Eq. 15. $$K_1(T) = exp\frac{-\Delta G_1(T)}{RT}$$

Eq. 16. $$K_2(T) = exp\frac{-\Delta G_2(T)}{RT}$$

Using analogous Gibbs-Helmholtz equations as for the two-state unfolding the measured total unfolded fraction $u = f_u(T)$ can be fit against the parameters of three-state unfolding via an intermediate state. The model can be easily transformed to three-state unfolding without an

intermediate state, supposing two independent unfolded states u_1 and u_2.

b. Structure calculations

MUMO ensemble. The MUMO ensemble was calculated with a modified version of GROMACS 4.5.5. that includes S2 order parameter restraining[7] and pairwise treatment of NOE restraints over the replicas[8]. Other aspects of NOE restraining were not modified relative to official GROMACS. The AMBER99SB force field was used with the explicit TIP4P water model. Structures were extracted every 200 ps from a 4 ns simulation with 16 replicas. By discarding conformers before the first 1 ns of the run, 256 structures were selected for the final ensemble. Correspondence to experimental data was verified using the CoNSEnsX web server[9].

First-approximation models for invisible states. For states not directly observable, a population selection approach was used. First, a conformer pool was generated using a modified version of GROMACS 4.5.5 with the accelerated molecular dynamics (AMD) scheme[10] implemented for the dihedral term. GROMACS source code files containig all of the modifications used are available at http://users.itk.ppke.hu/~gaszo. For the pool generation, the GBSA implicit water model using the Onufriev-Bashford-Case method[11] to calculate the Born radii was used. Chemical shifts for all conformers were estimated with SHIFTX2[12]. In the next step, a random selection algorithm was used to select a sub-ensemble that corresponds to the observed/deduced chemical shifts of the given state. Instead of a fixed size for the final ensemble, only a minimal size of 2 for the target ensemble was used. After randomly selecting an initial set of conformers, eliminations and additions from the pool were performed while requiring that the agreement with experimental data increases at each step, and a maximum of 10,000 steps were allowed. Agreement with experimental chemical shifts was monitored by calculating either simple Pearson correlation or Q-factor. The selection procedure was repeated 10,000 times, and the conformers most often selected were listed. The final representative first-approximation models were selected upon inspection and comparison of these lists for different states, by seeking the consensus of the correlation and Q-factor-selected ensembles.

Eq. 17. $$\Delta V(r) = \frac{[E - V(r)]^2}{\propto + [E - V(r)]}$$

Calculation of the modified potential according to the AMD scheme. *E* denotes the boost energy threshold below which no modification is performed.

For the high- and low-temperature states, the AMD calculations were run with a setup allowing moderate sampling of the conformational space (dihedral boost energy: 5000 kJ/mol, alpha: 100). A total of 2001 models were generated including the starting model (corresponding to the native structure) by extracting conformations every 20 ps from a 40 ns run. The representative models

were selected by listing the conformers showing the largest difference in their selection frequency in the 2x10,000 random selection runs. For these selections, both ^{15}N and $^{13}C\alpha$ shifts were used.

To obtain approximate structures for the conformers of the 'visible portion' selected from all PAF states as a function of temperature, we used an ensemble selection-based approach. Observation of the chemical shifts at the two extreme temperatures (268K and 344K) led us to the following conclusions: 1) the observable hot and cold states are distinct from each other and from the "native" conformation (2mhv) at ambient temperature (298K) and 2) the magnitude of possible structural changes is moderate, complete unfolding/restructuring is not reasonable. Thus, we generated a structure pool using the accelerated molecular dynamics scheme with acceleration parameters allowing the exploration of the conformational space in the neighborhood of the native conformational ensemble. Accordingly, for these states we assume that the "abcabc" disulfide pattern remains intact (no rearrangement) - and this is a serious restriction for the conformations generated - though individual disulfide bond conformations were allowed to change. The selection approach utilized the conformationally most sensitive experimental CA and HA shifts besides the available amide N and H shifts, and the size of the ensemble was not restricted. The resulting ensembles were typically small, consisting of only 3-4 structures. The disulfide constraints render overfitting unlikely (agreement between observed and back-calculated chemical shifts is the result of averaging only between allowed conformers). This is supported by the fact that many of these ensembles include the starting conformer of the AMD simulation which is closest to the 'native' (ambient-temperature) state, thus, these ensembles are expected to retain native-like conformers. As we were interested in the differences between the high and low-temperature states, we performed 10,000 runs of ensemble generation at both temperatures and have chosen the conformers with the largest differences in their occurrence in the two ensembles. Thus, the native like conformers at low and high temperatures shown and discussed in the manuscript (Fig 6.) are indicative for the *differences* between these states, and do not represent the full ensemble that involves other interconverting, invisible conformers. Accordingly, the present approach, though limited, is still a suitable way for obtaining approximate structural representation in agreement with the observed chemical shift differences at low and high temperatures.

For the hidden state detected by the CEST experiment, a more aggressive sampling was performed (dihedral boost energy: 5000 kJ/mol, alpha: 100), requiring that the individual extracted conformations were energy-minimized before SHIFTX2 calculations in order to clean up unfavorable geometries. The list of experimental shifts of the hidden state were compiled using the observed shifts modified by the CEST-detected offset for the residues affected. Conformers with best correspondence to these ^{15}N chemical shifts deduced for the hidden state were selected as approximate models. It should be noted that these models are based on limited chemical shift information and that structural differences at sites where CEST did not reveal conformational exchange can not be regarded as reliable. Principal component analysis was performed with ProDy[13] .

II. Supplementary results

a. Results of structure refinement

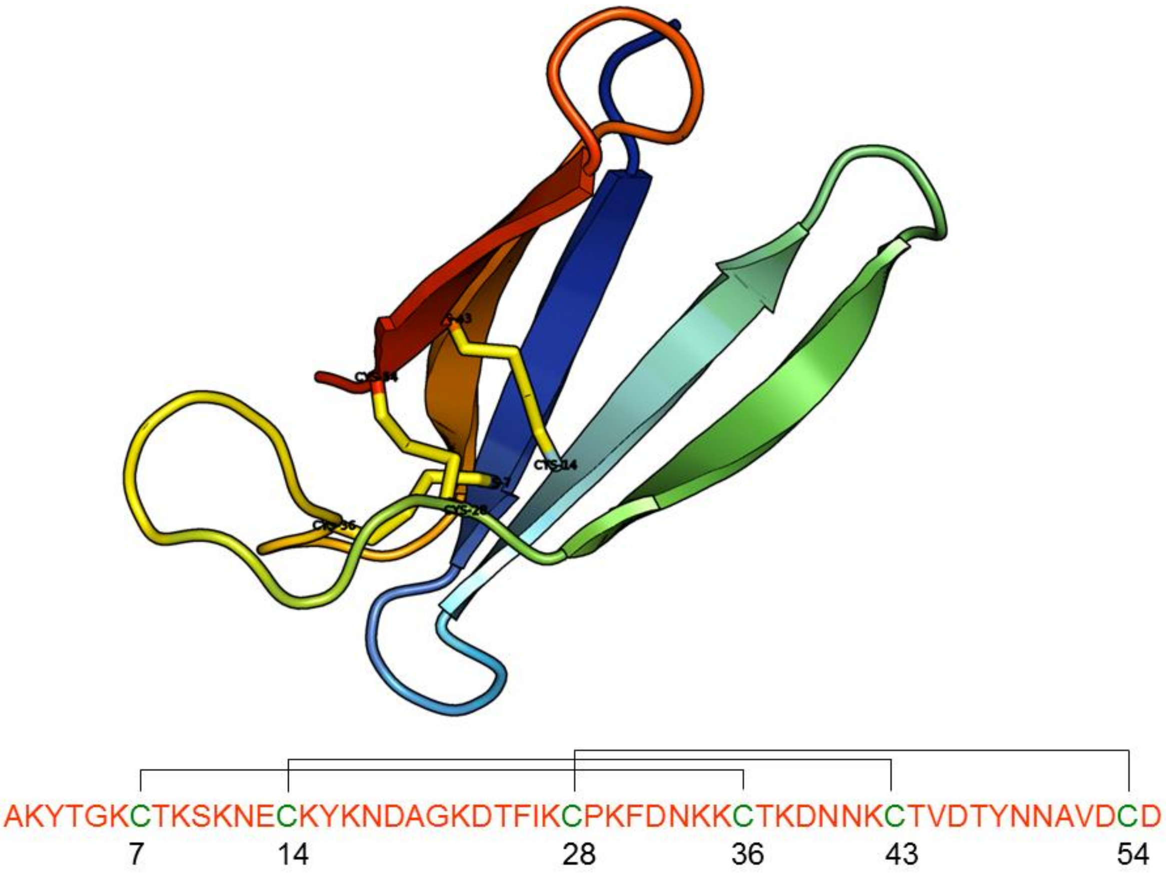

Figure 1.: refined PAF structure with explicit disulfide bonds (pdb code:2mhv)

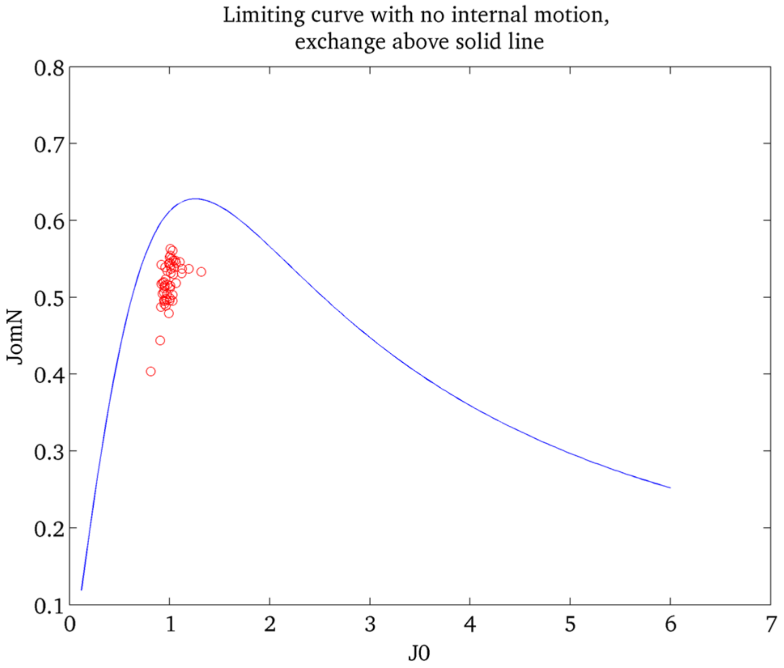

Figure 1.: Reduced spectral density mapping of PAF at 304K (^{15}N-relaxation data T_1, T_2 and NOE are taken from[14]). The limiting curve accords to the global correlation time (τc = 3ns) from Lipari-Szabo analysis[15]. No signs of exchange at ps/ns timescale can be seen.

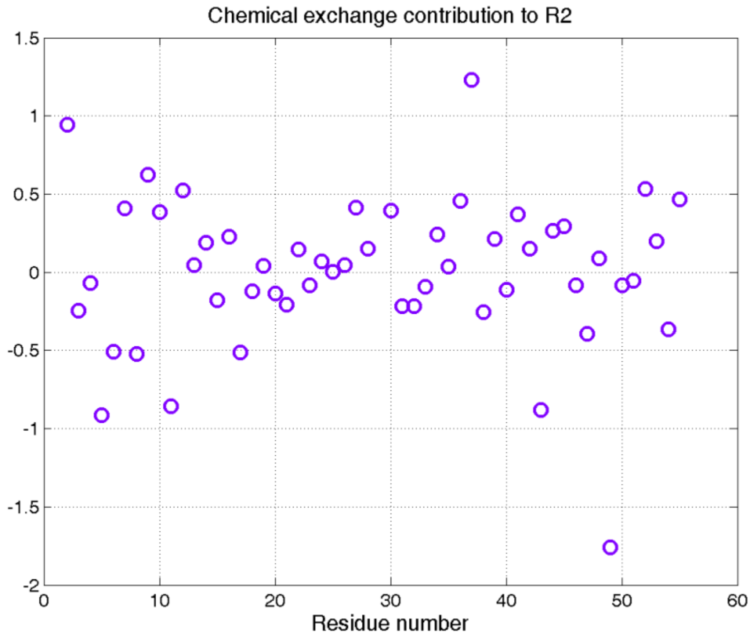

Figure 2.: Chemical exchange contribution to R_2 transverse relaxation rates of PAF derived from ^{15}N relaxation of PAF, including ^{15}N relaxation interference terms (data measured at 304K from[14] were used). Evaluation is according to Kroenke[16] that yields the average exchange contribution as R_{ex} = 0.0 ± 0.5 s-1

$R_{ex} = R_2 - R_2^o$ and $R_2^o = (R_1 - 1.249\ \sigma_{NH})\ \eta_{xy}/\eta_z + 1.079\ \sigma_{NH}$

Successful two-state fits of unfolding for NH crosspeak integrals (Evaluation of temperature dependence of ^{15}N-HSQC experiments):

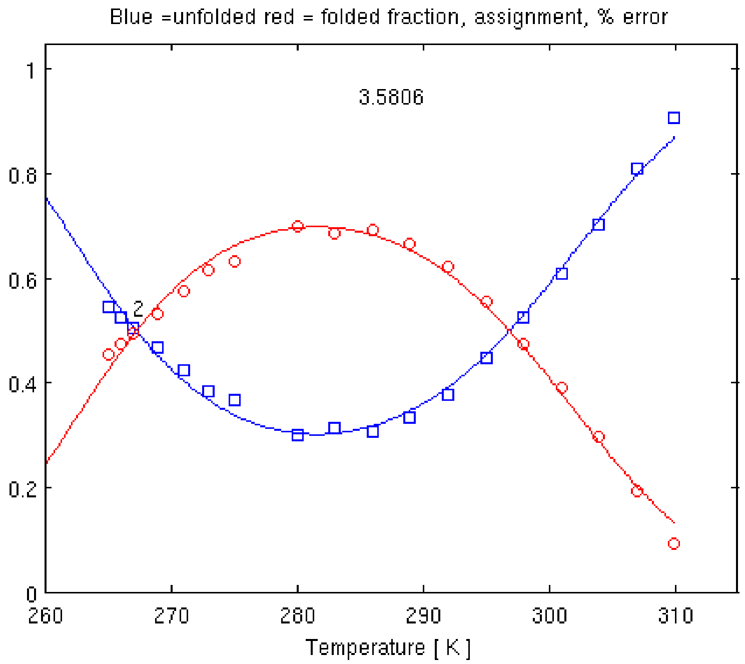

Figure 3.: Fit of Lys-2 unfolding using two-state model

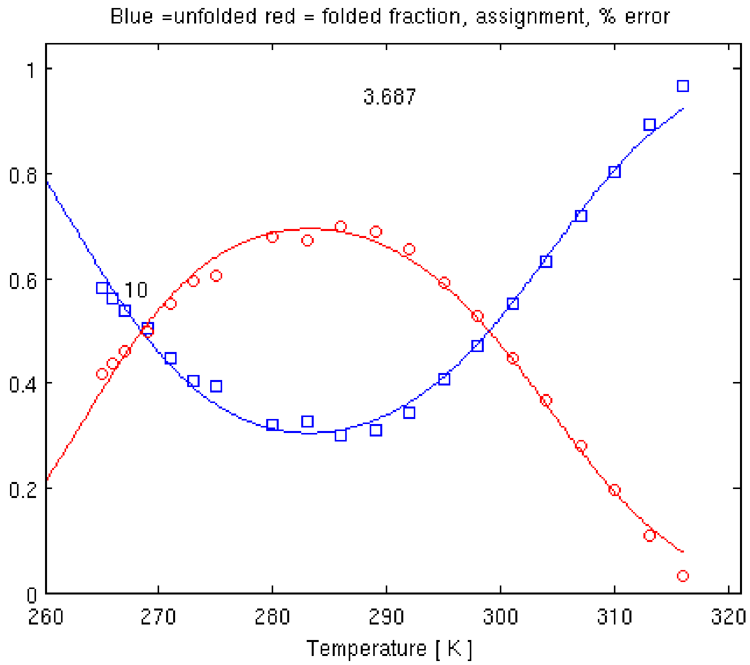

Figure 4.: Fit of Ser-10 unfolding using two-state model

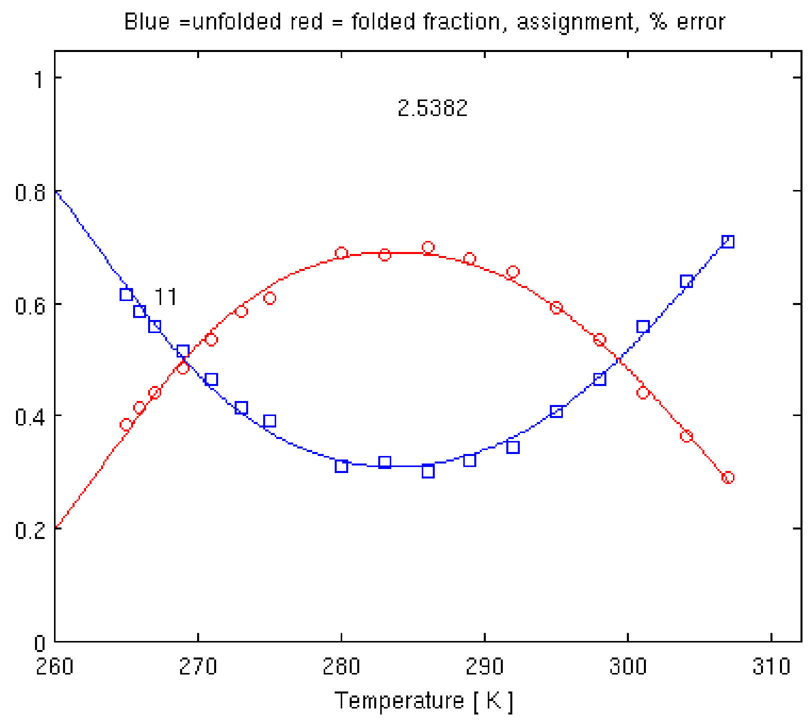

Figure 5.: Fit of Lys-11 unfolding using two-state model

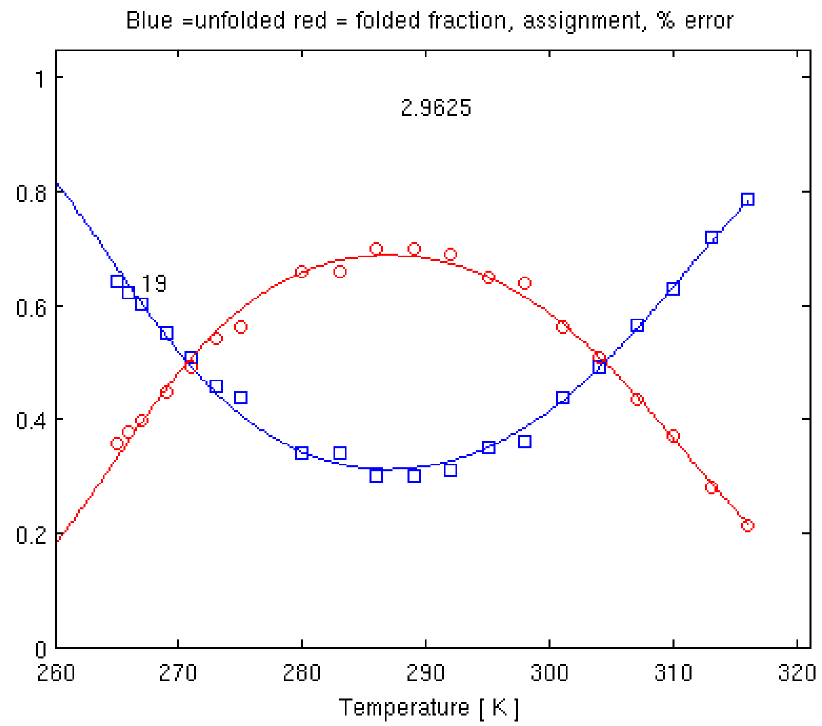

Figure 6.: Fit of Asp-19 unfolding using two-state model

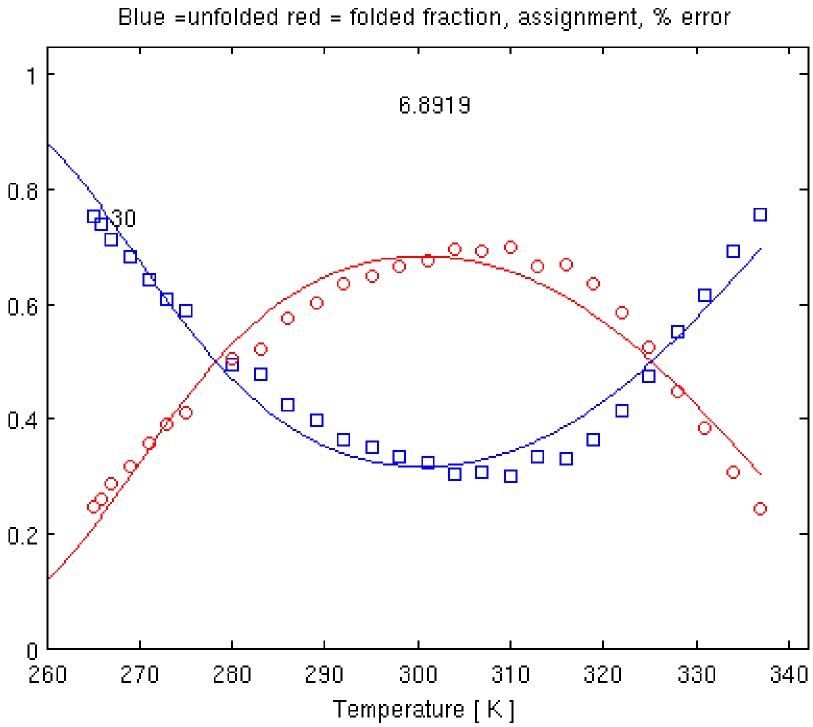

Figure 7.: Fit of Lys-30 unfolding using two-state model

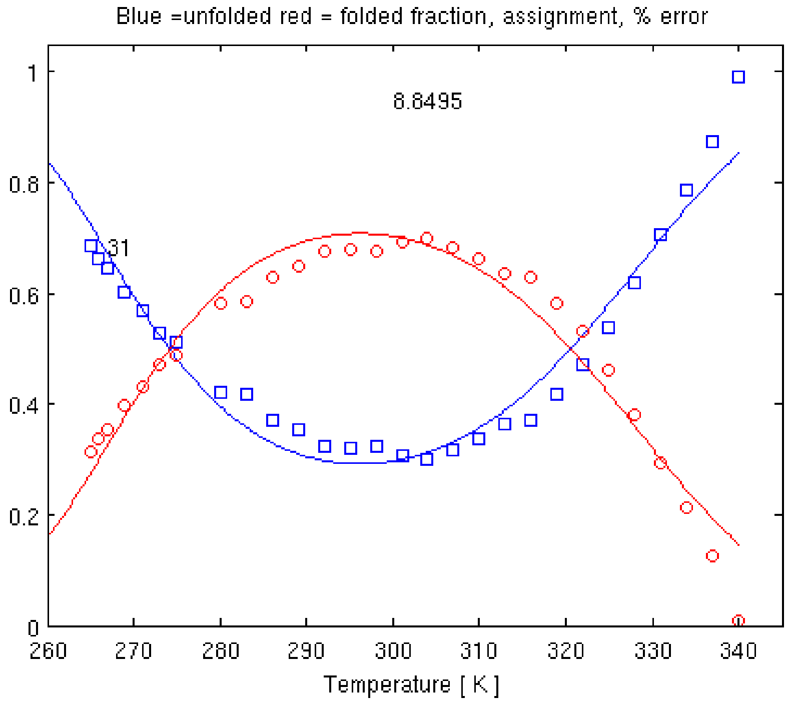

Figure 8.: Fit of Phe-31 unfolding using two-state model

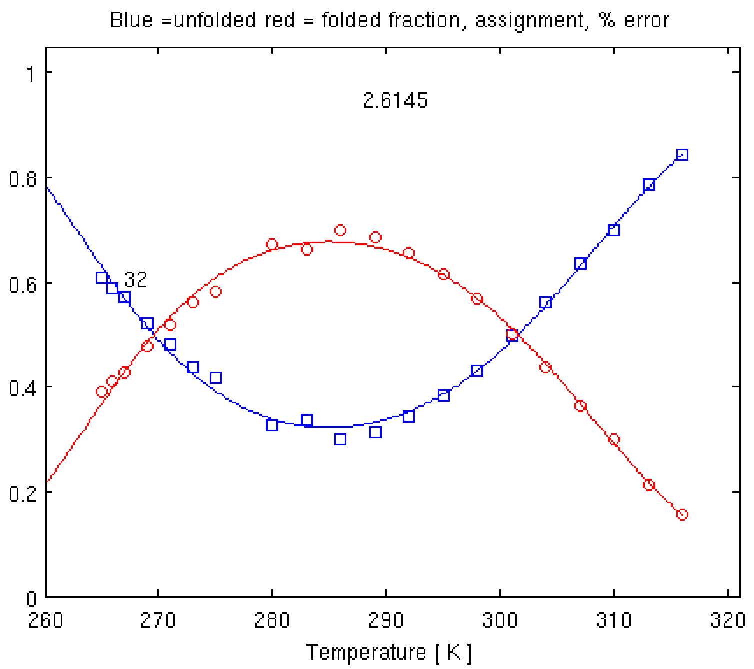

Figure 9.: Fit of Asp-32 unfolding using two-state model

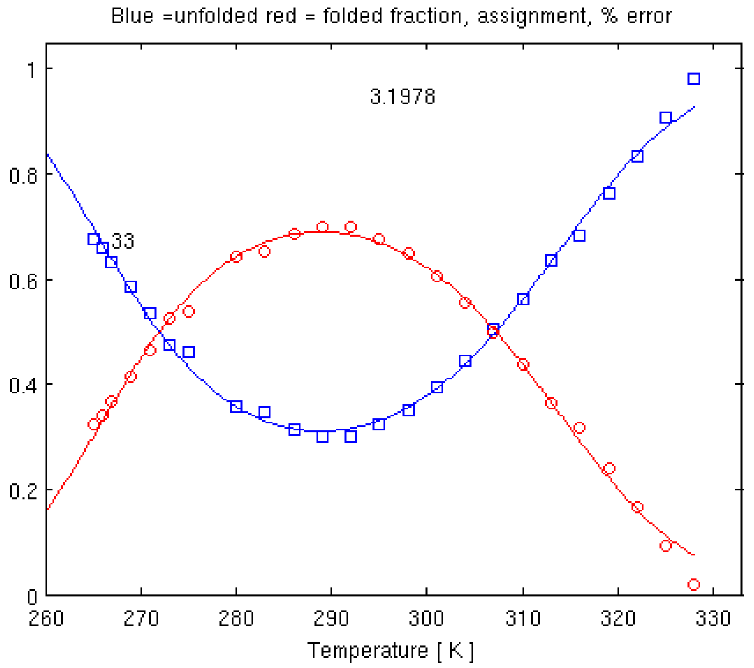

Figure 10.: Fit of Asn-33 unfolding using two-state model

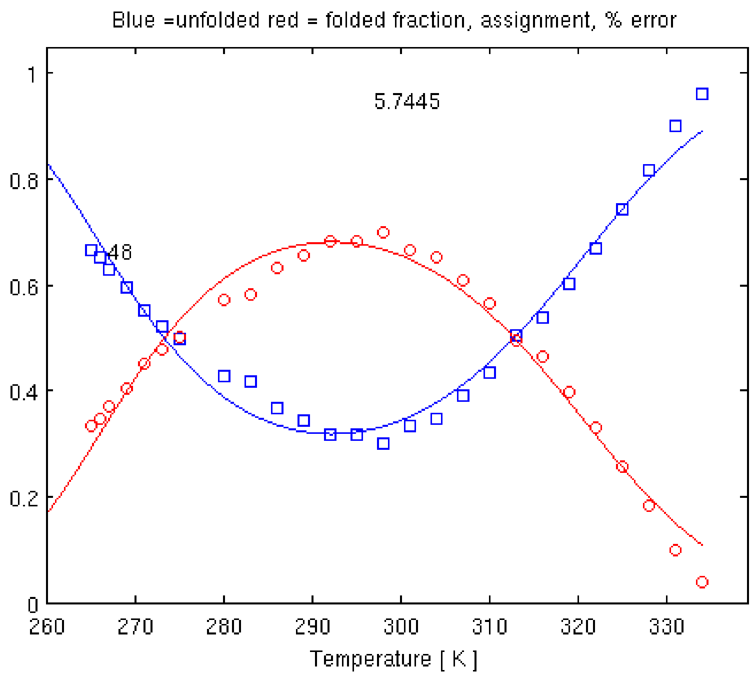

Figure 11.: Fit of Tyr-48 unfolding using two-state model

Analysis of the thermal unfolding fits using two- and three-state unfolding models:

Group-1 residues: K2, S10, K11, D19, K30, F31, D32, N33, Y48

Group-2 residues: 3 4 5 7 8 9 13 14 16 18 20 21 24 25 26 27 34 36 37 38 39 40 42 44 47 50 51 54 55

Table 1.

Averages of two-state model fitting based on temperature dependent ^{15}N-HSQC peak integrals. Results obtained after applying the "PULCON" protocol for raw data are labelled with asterisk*.

	ΔH_u kJ/M	T_{high} / K	T_{low} / K	$T_{maxstab}$ / K	ΔC_p kJ/M/K	ΔG_{u_max} kJ/M	Error (%)
Group-1	65 ± 7	305 ± 10	267 ± 3	286 ± 6	3.5 ± 1.0	2.0 ± 0.2	3 ± 1
*	66 ± 9	307 ± 10	271 ± 4	289 ± 7	3.7 ± 1.1	1.9 ± 0.1	4 ± 2
Group-2	55 ± 4	328 ± 4	272 ± 3	300 ± 3	1.9 ± 0.2	2.4 ± 0.2	13 ± 3
*	46 ± 5	331 ± 5	281 ± 4	306 ± 4	1.8 ± 0.3	1.8 ± 0.2	15 ± 3

Table 2.

Averages of two-state model fitting based on temperature dependent ^{13}C-CT-HSQC peak integrals

	ΔH_u kJ/M	T_{high} / K	T_{low} / K	$T_{maxstab}$ / K	ΔC_p kJ/M/K	ΔG_{u_max} kJ/M	Error (%)
Group-1	35 ± 11	329 ± 5	290 ± 10	309 ± 15	1.8 ± 0.4	1.1 ± 0.5	11 ± 2
Group-2	36 ± 10	330 ± 16	292 ± 5	310 ± 21	2.0 ± 0.5	1.1 ± 0.5	10 ± 2

Table 3a.

Averages of three-state model (FIU, F-I transition) fitting based on temperature dependent ^{15}N-HSQC peak integrals. Results obtained after applying the "PULCON" protocol for raw data processing (labelled with asterisk*).

	ΔH_{u1} kJ/M	T_{high1} / K	T_{low1} / K	$T_{maxstab}$ / K	ΔC_{p1} kJ/M/K	ΔG_{u1_max} kJ/M	Error %
Group-1	71 ± 19	318 ± 35	246 ± 35	278 ± 70	2.4 ± 1.3	4.3 ± 3.1	1.6 ± 0.6
*	65 ± 13	353 ± 39	271 ± 5	312 ± 44	1.8 ± 1.1	3.8 ± 1.6	2.1 ± 0.6
Group-2	38 ± 4	347 ± 9	270 ± 6	308 ± 15	0.9 ± 0.2	2.2 ± 0.1	2.9 ± 0.8
*	34 ± 3	392 ± 12	284 ± 5	338 ± 16	0.5 ± 0.2	2.7 ± 0.4	2.6 ± 0.6

Table 3b.

Averages of three-state model (FIU, I-U transition) fitting based on temperature dependent ^{15}N-HSQC peak integrals

	ΔH_{u2} kJ/M	T_{high2} / K	T_{low2} / K	$T_{maxstab}$ / K	ΔC_{p2} kJ/M/K	Error %
Group-1	176 ± 196	334 ± 37	246 ± 35	290 ± 72	2.6 ± 2.5	1.6 ± 0.6
Group-2	273 ± 78	334 ± 3	218 ± 48	276 ± 51	3.7 ± 3.0	2.9 ± 0.8

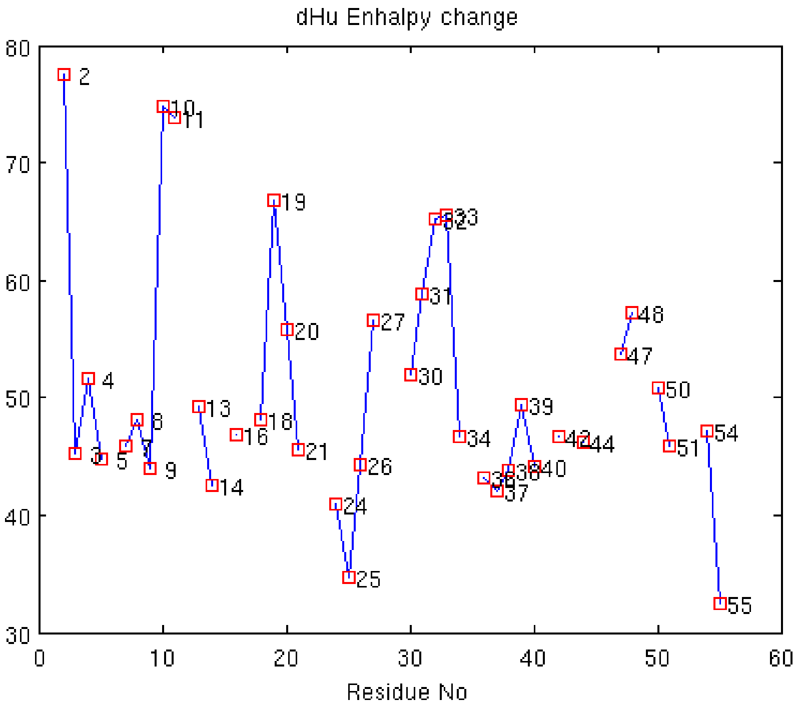

Figure 13: Residue specific ΔHu enthalpy change of unfolding (kJ/M) using two-state model (NH-peaks).

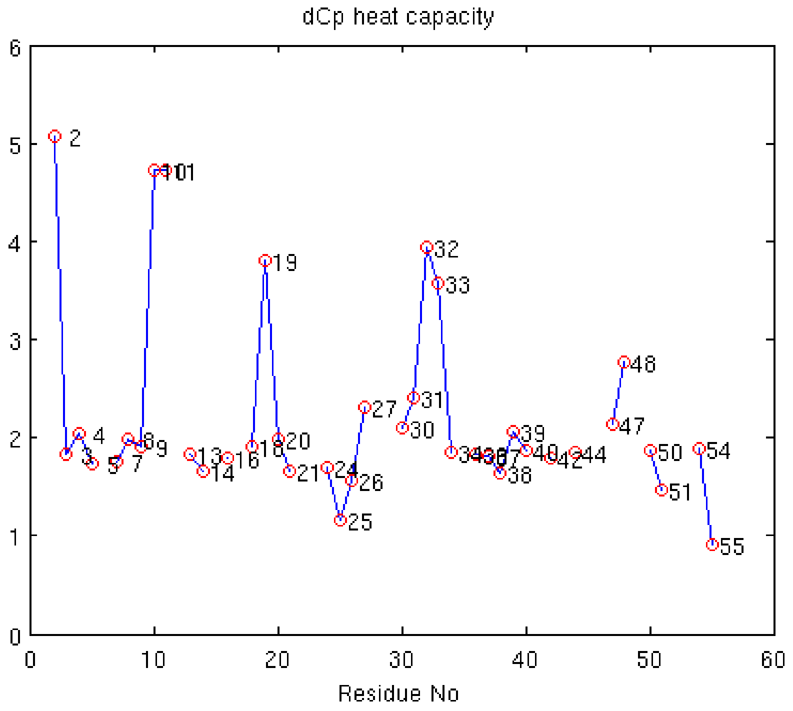

Figure 14: Residue specific ΔCp enthalpy change of unfolding (kJ/M/K) using two-state model (NH-peaks).

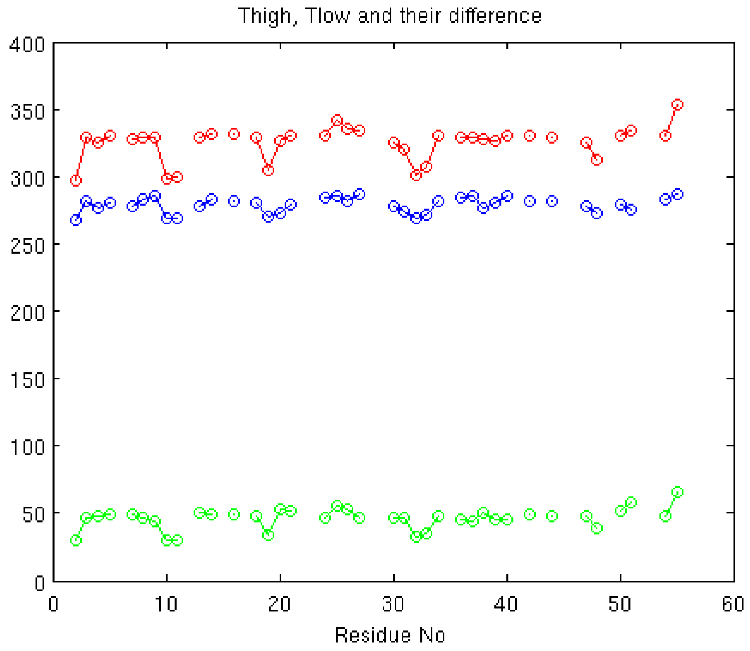

Figure 15: Residue specific high(red)-and low(blue) temperature melting points (K) using two-state model (NH-peaks). Cold unfolding is concerted in contrast to heat unfolding.

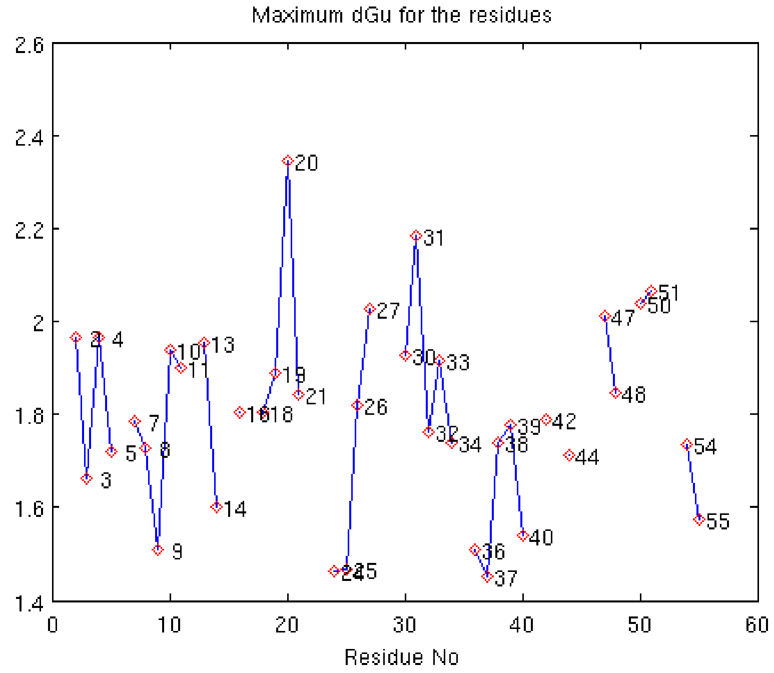

Figure 16: Residue specific maxima of the ΔGu free-enthalpy change of unfolding (kJ/M) using two-state model (NH-peaks).

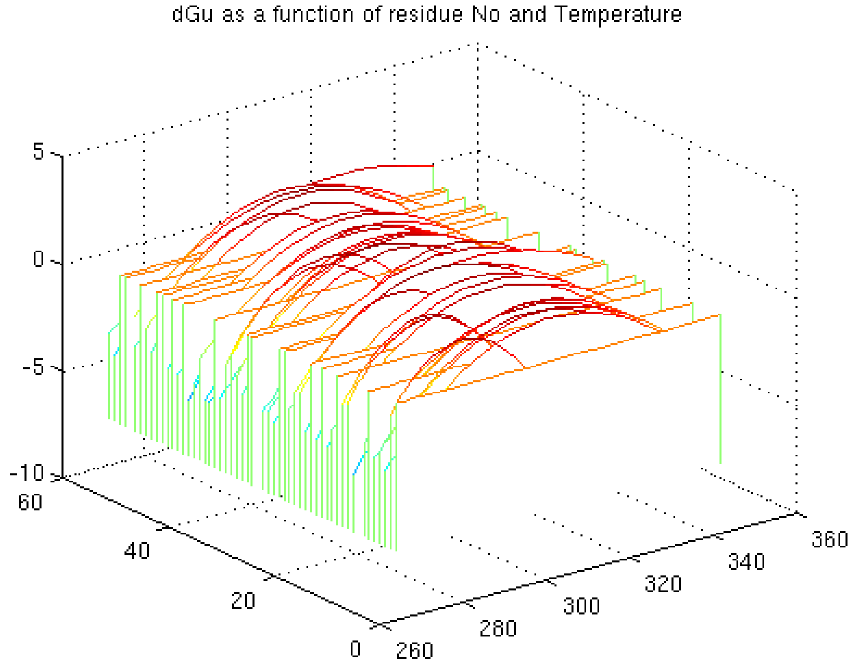

Figure 17: Residue specific ΔGu free-enthalpy change of unfolding (kJ/M) as a function of temperature (K) using two-state model (NH-peaks).

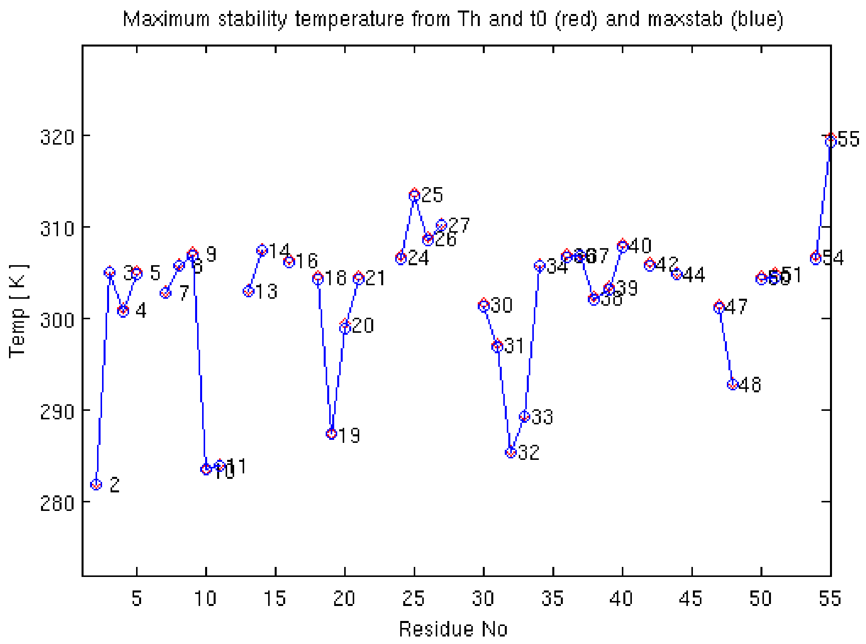

Figure 18: Residue specific maximum stability temperatures in PAF using two-state model (NH-peaks).

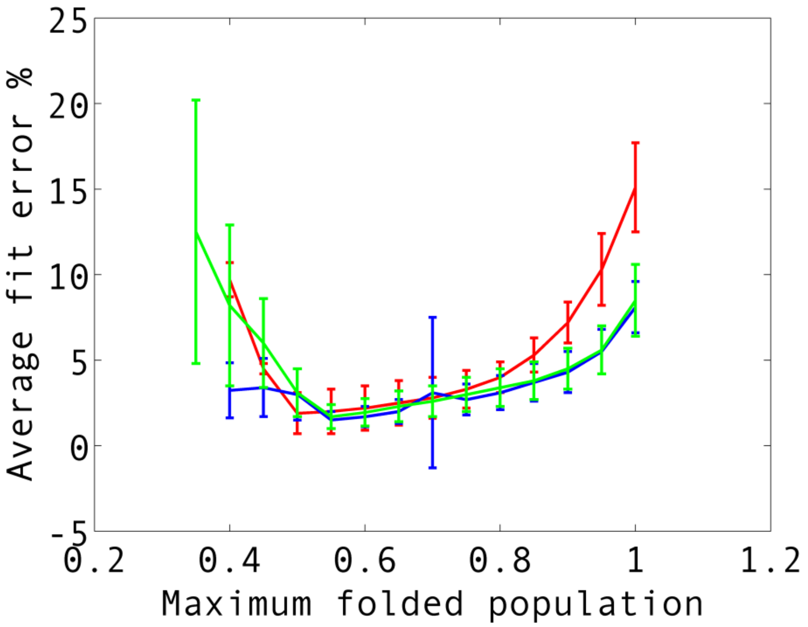

Figure 19: Global fitting errors as a function of maximum stability populations from ^{15}N-HSQC spectra (FU (red), FIU(green) and FU2 (blue) models)

Two-state fits of unfolding for some ^{13}Cα–Hα crosspeak integrals (results of CT-^{13}C-HSQC experiments using ^{15}N/^{13}C labelled PAF):

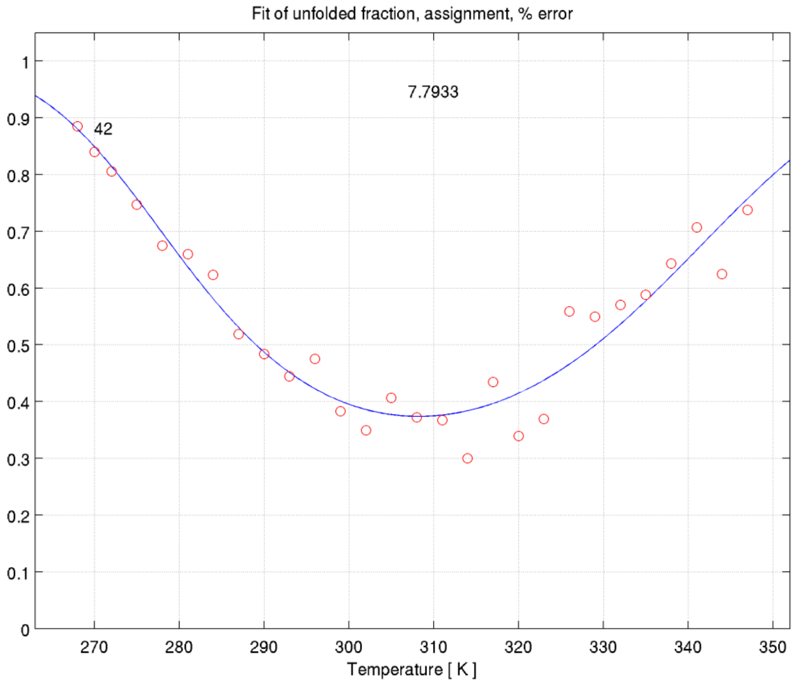

Figure 20 : Two-state fit of unfolding of residue 42 for ^{13}Cα-Hα crosspeaks

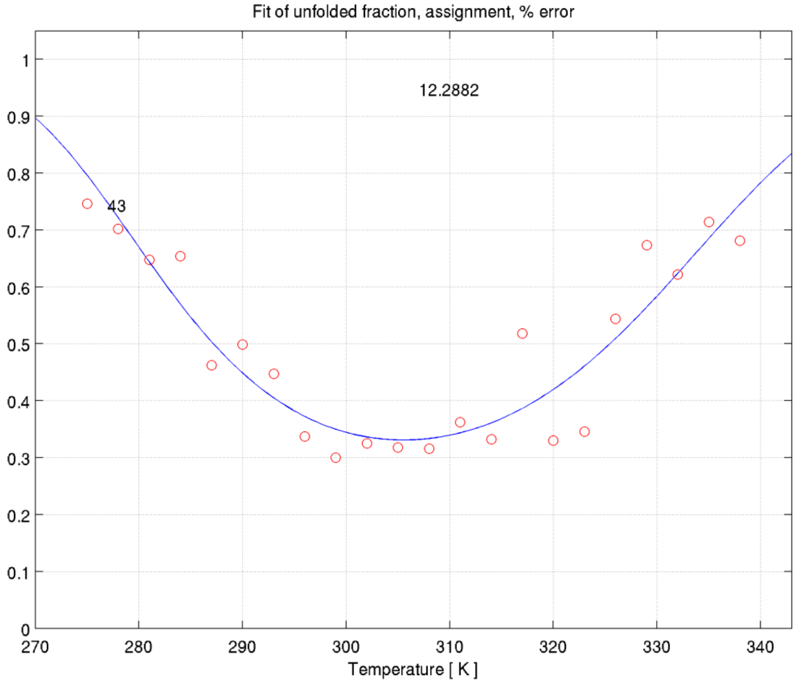

Figure 21: Two-state fit of unfolding of residue 43 for ^{13}Cα-Hα crosspeaks

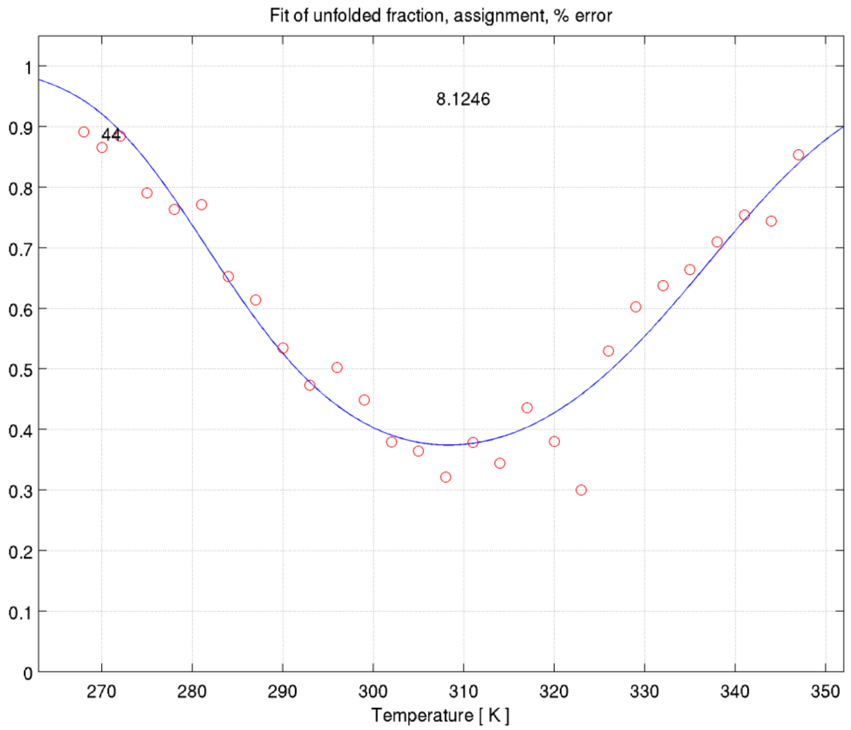

Figure 22: Two-state fit of unfolding of residue 44 for ^{13}Cα-Hα crosspeaks

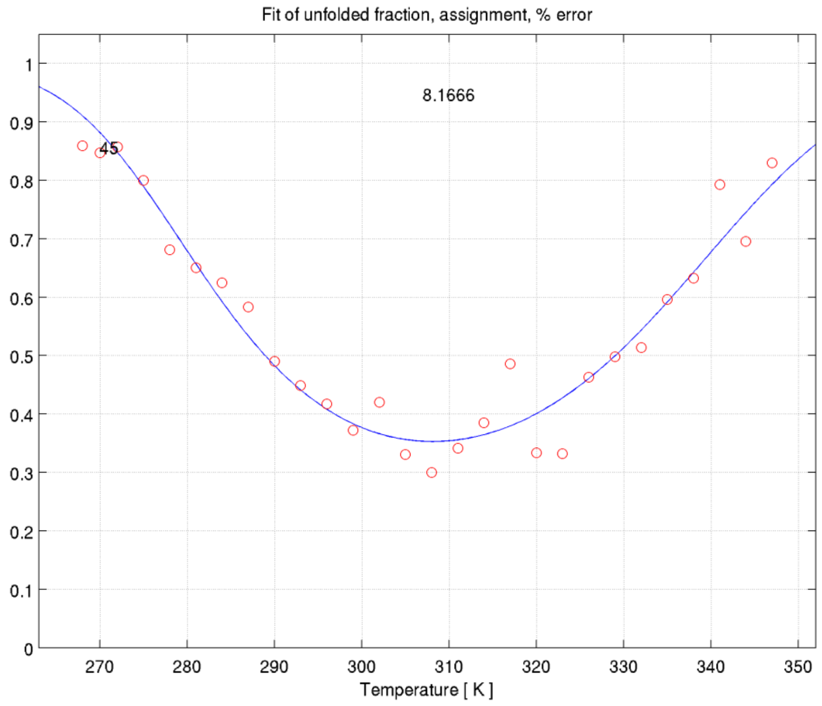

Figure 23: Two-state fit of unfolding of residue 45 for ^{13}Cα-Hα crosspeaks

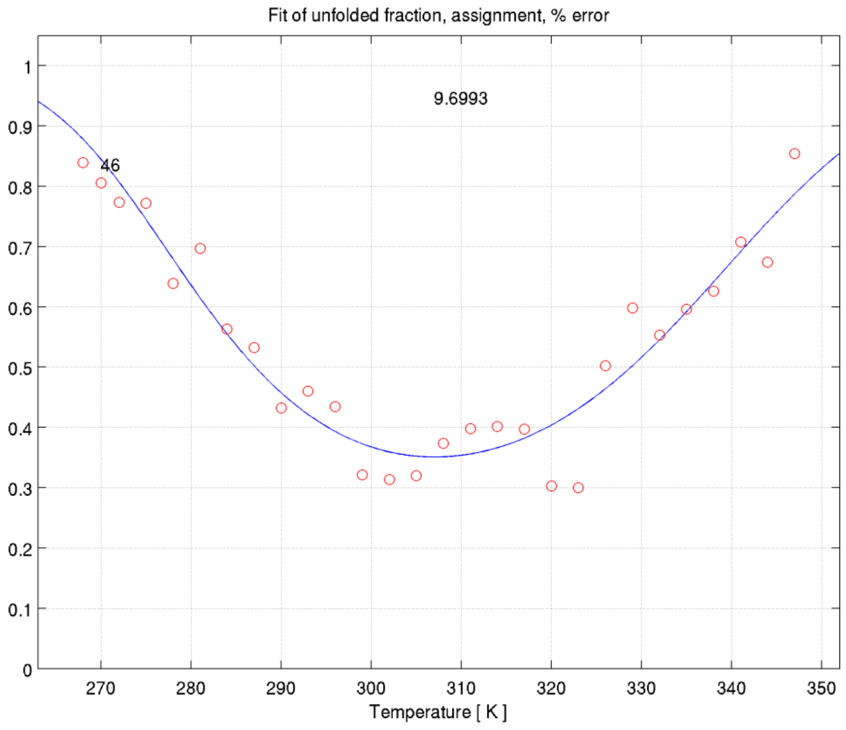

Figure 24: Two-state fit of unfolding of residue 46 for $^{13}C\alpha$-Hα crosspeaks

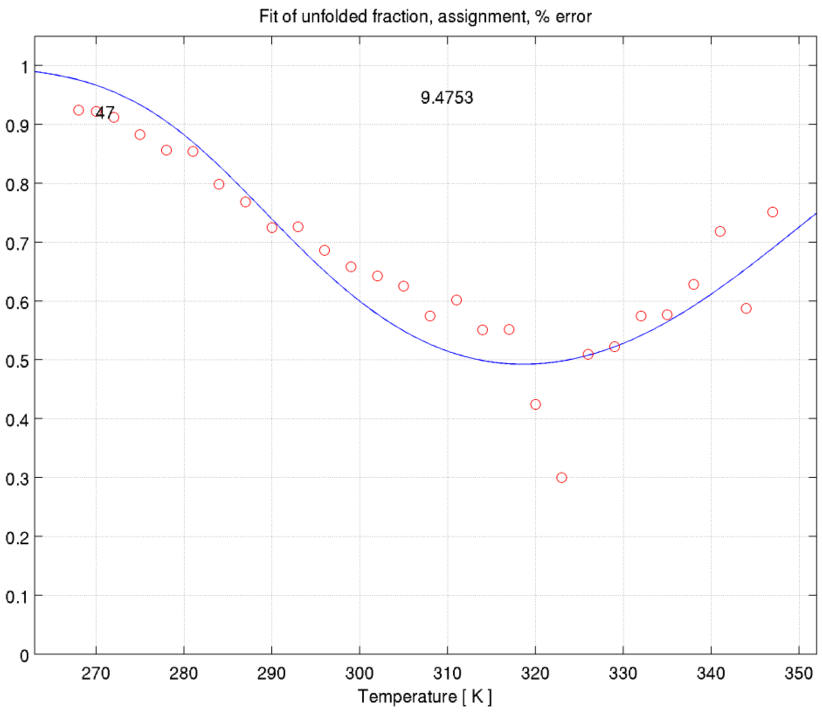

Figure 25: Two-state fit of unfolding of residue 47 for $^{13}C\alpha$-Hα crosspeaks

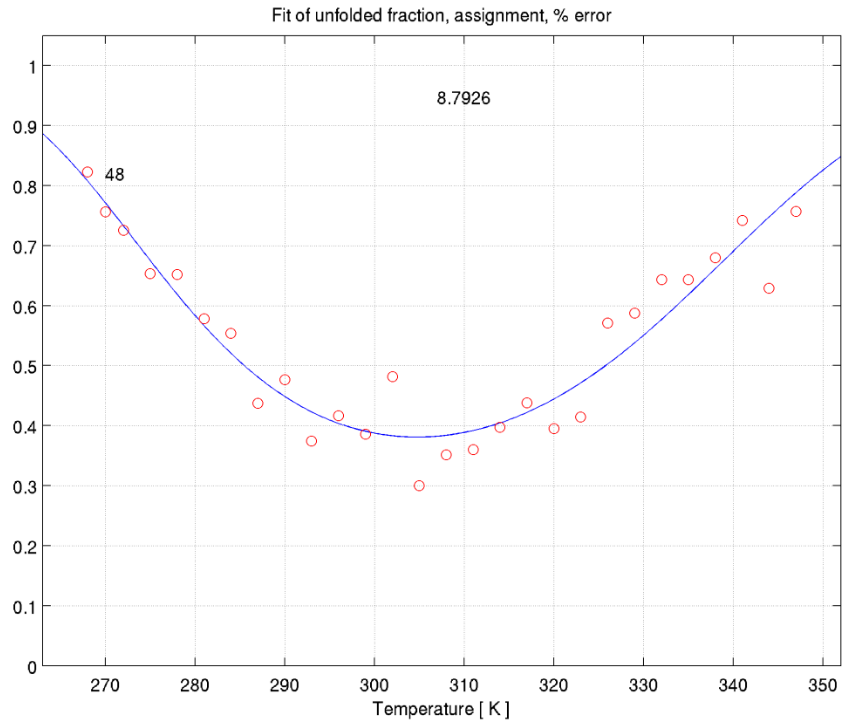

Figure 26: Two-state fit of unfolding of residue 48 for $^{13}C\alpha$-Hα crosspeaks

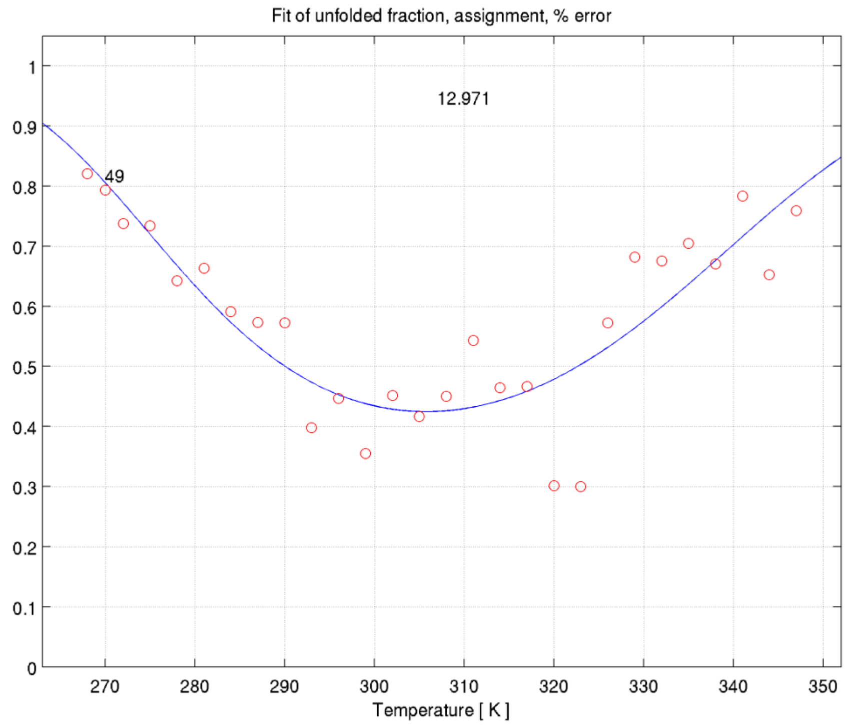

Figure 27: Two-state fit of unfolding of residue 49 for $^{13}C\alpha$-Hα crosspeaks

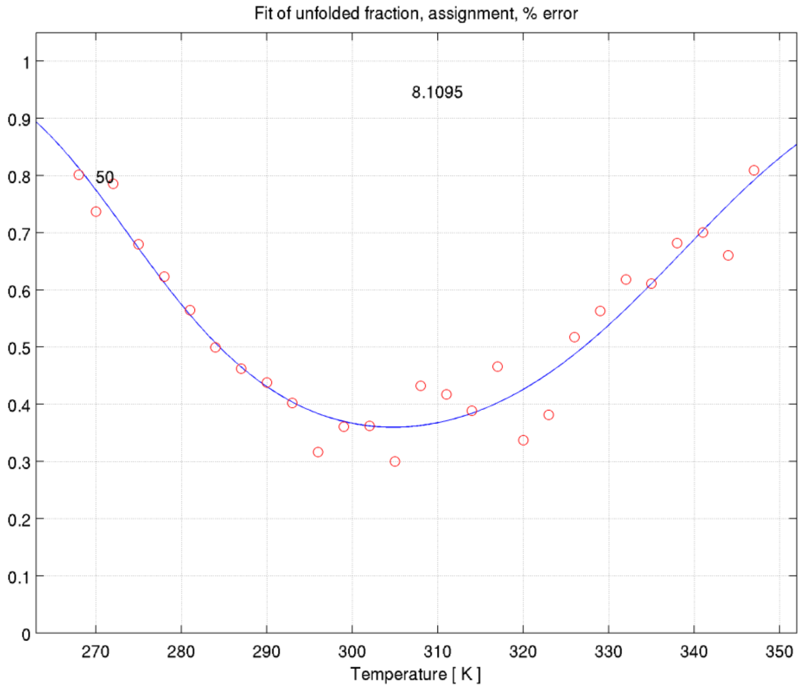

Figure 28: Two-state fit of unfolding of residue 50 for $^{13}C\alpha$-Hα crosspeaks

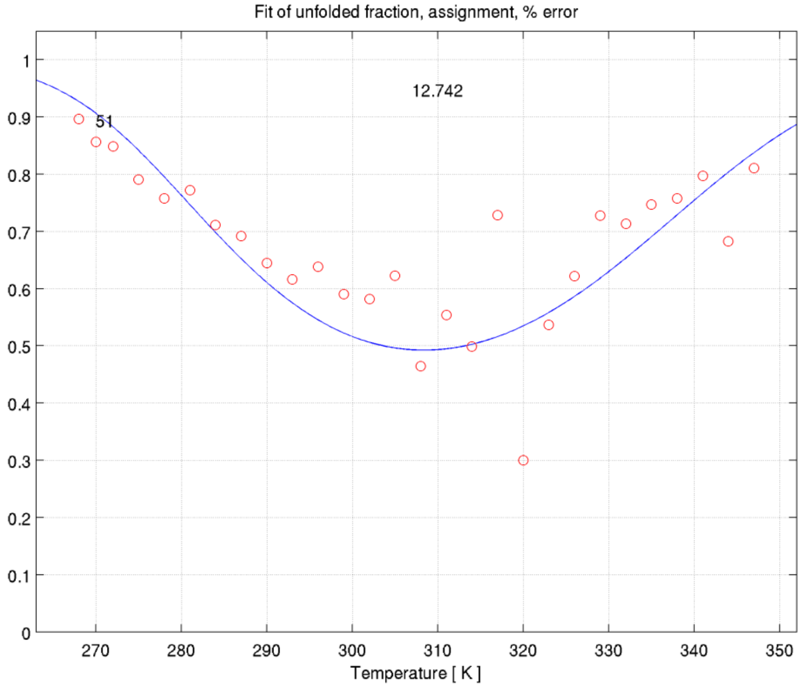

Figure 29: Two-state fit of unfolding of residue 51 for $^{13}C\alpha$-Hα crosspeaks

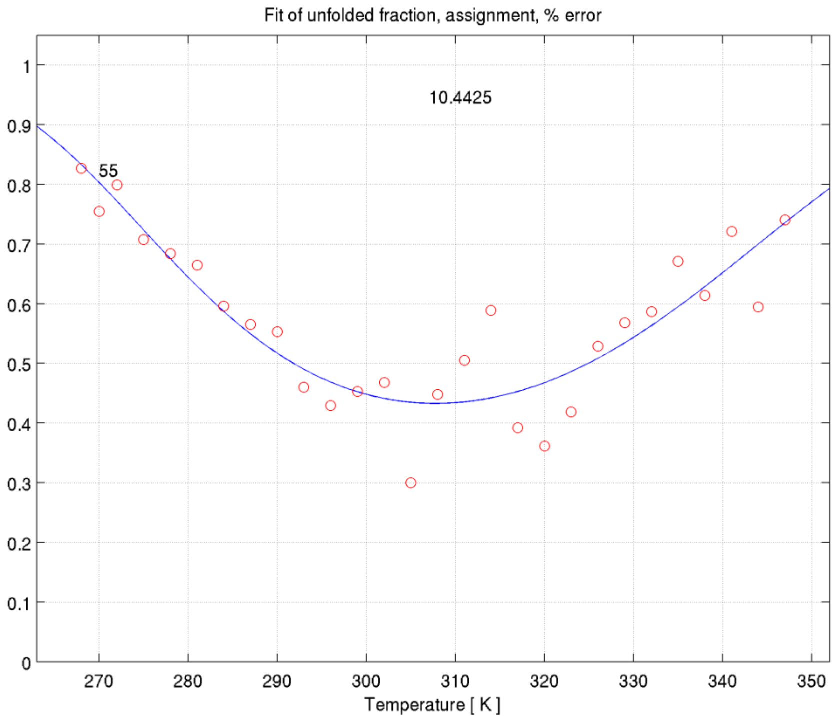

Figure 30: Two-state fits of unfolding of residue 55 for $^{13}C\alpha$-Hα crosspeaks

Examples of fitting the measured chemical shifts:

The ^{15}N shifts are fit against the measured folded F and unfolded U populations, while the combined NH-shifts are fit against the populations F, U1 and U2 calculated from the FIU model. A conformational drift model is supposed causing linear temperature drifts of the putative chemical shifts of each conformer. The measured chemical shift is calculated as a population weighted average of the putative chemical shifts of each conformers.

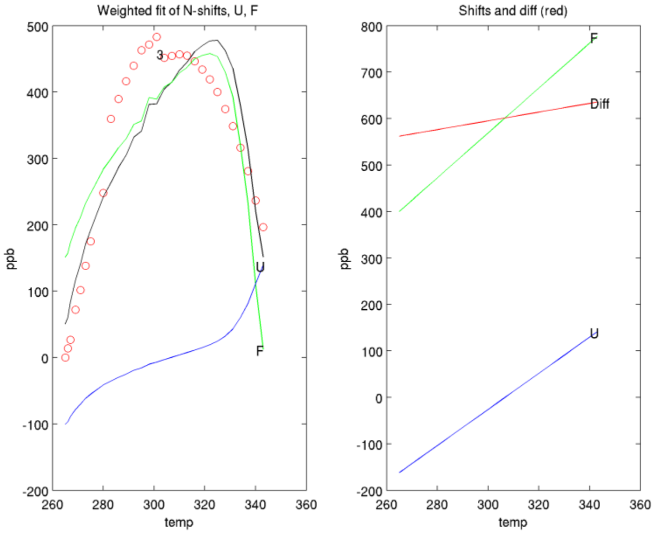

Figure 31: ^{15}N chemical shift fit of Tyr-3 based on the populations of folded (F) and unfolded (U) conformers. Left panel with red “o” shows fit (black line) of experimental shifts, right panel displays putative ^{15}N shifts.

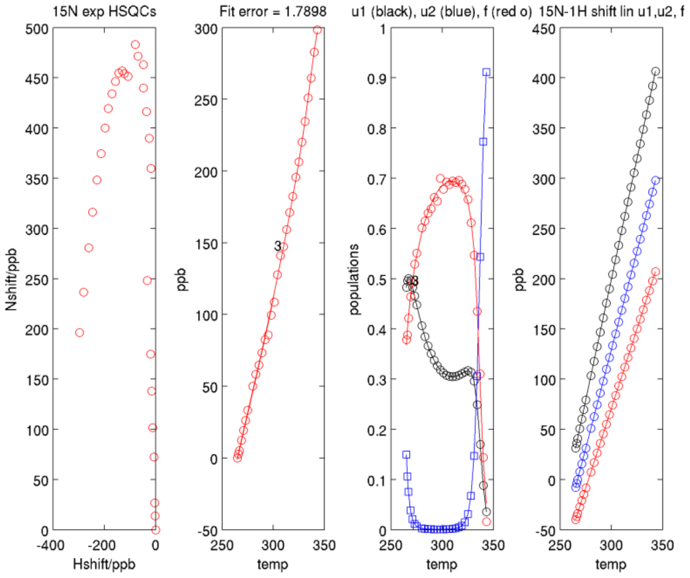

Figure 32: FIU model fit of Tyr-3 (panels from left to right: 1. NH-shifts in HSQC spectra, 2. fit of combined NH-shift, 3. populations f, u1 and u2, 4. drift of putative chemical shifts)

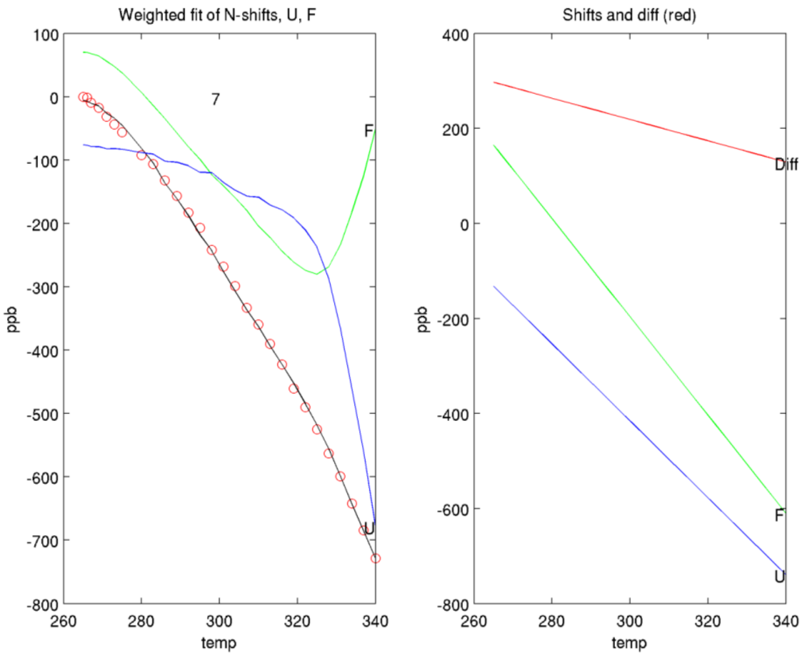

Figure 33: ^{15}N chemical shift fit of Cys-7 based on the populations of folded (F) and unfolded (U) conformers. Left panel with red "o" shows fit (black line) of experimental shifts, right panel displays putative ^{15}N shifts.

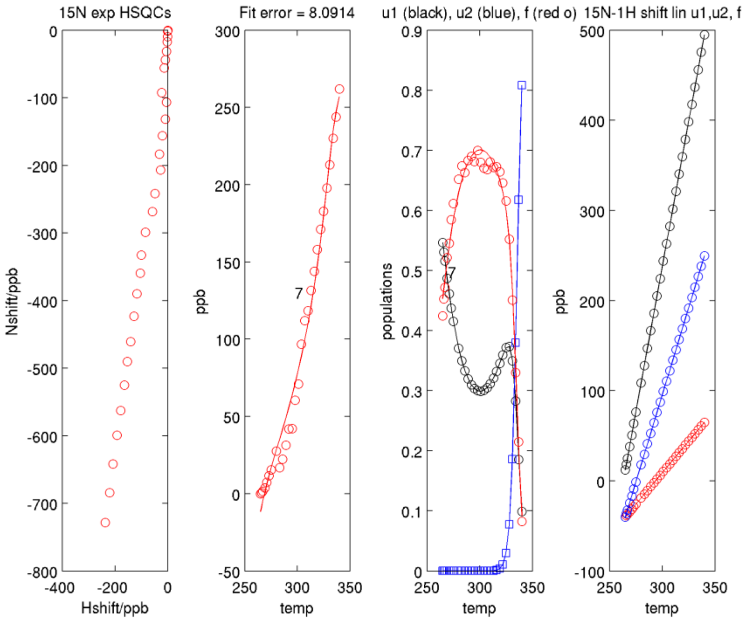

Figure 34: FIU model fit of Cys-7 (panels from left to right: 1. NH-shifts in HSQC spectra, 2. fit of combined NH-shift, 3. populations f, u1 and u2, 4. drift of putative chemical shifts)

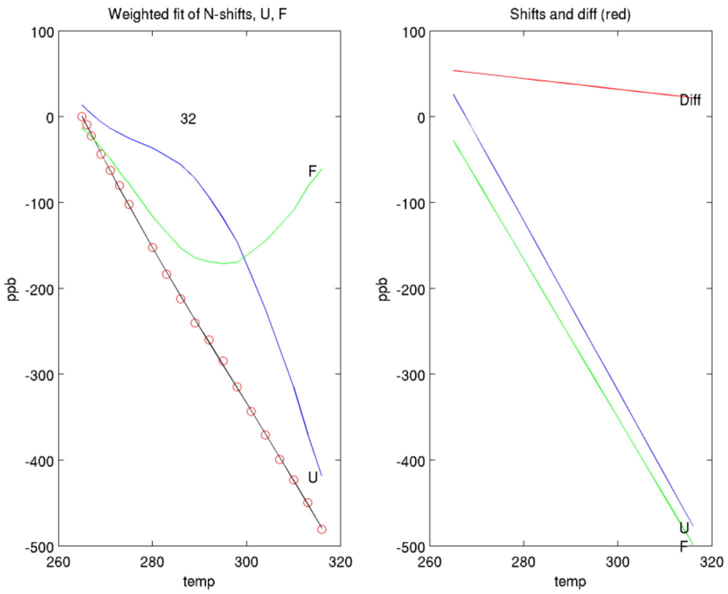

Figure 35: [15]N chemical shift fit of Asp-32 based on the populations of folded (F) and unfolded (U) conformers. Left panel with red “o” shows fit (black line) of experimental shifts, right panel displays putative ^{15}N shifts.

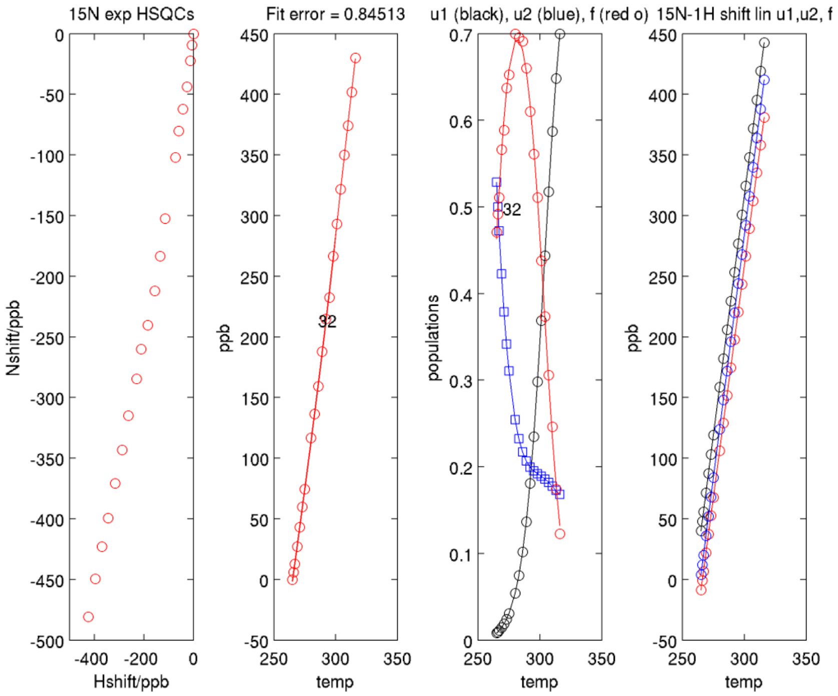

Figure 36: FIU model fit of Asp-32 (panels from left to right: 1. NH-shifts in HSQC spectra, 2. fit of combined NH-shift, 3. populations f, u1 and u2, 4. drift of putative chemical shifts)

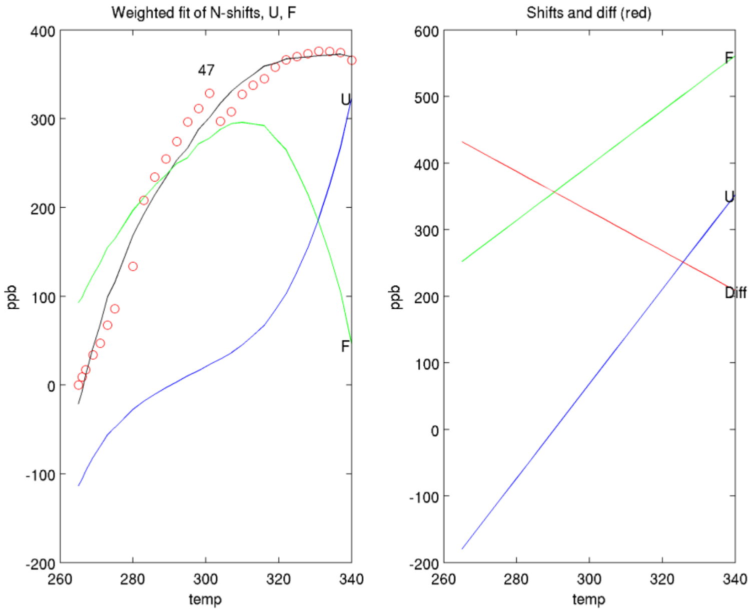

Figure 37: ^{15}N chemical shift fit of Thr-47 based on the populations of folded (F) and unfolded (U) conformers. Left panel with red "o" shows fit (black line) of experimental shifts, right panel displays putative ^{15}N shifts.

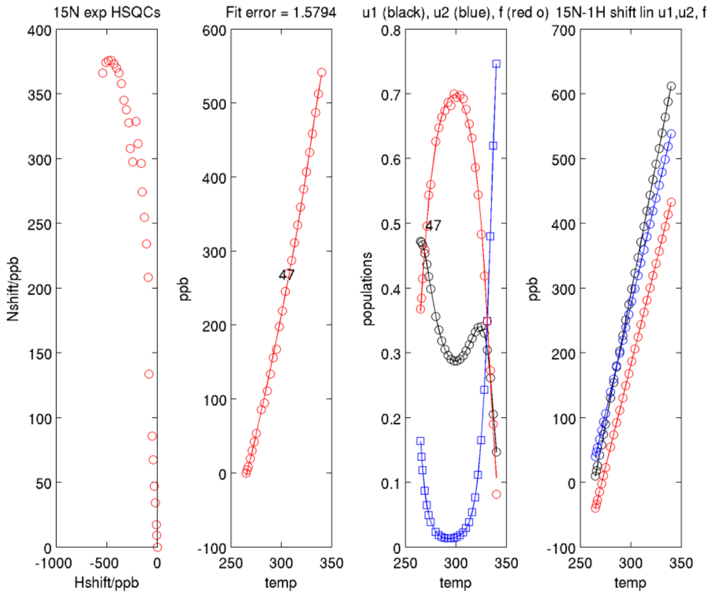

Figure 38: FIU model fit of Thr-47 (panels from left to right: 1. NH-shifts in HSQC spectra, 2. fit of combined NH-shift, 3. populations f, u1 and u2, 4. drift of putative chemical shifts)

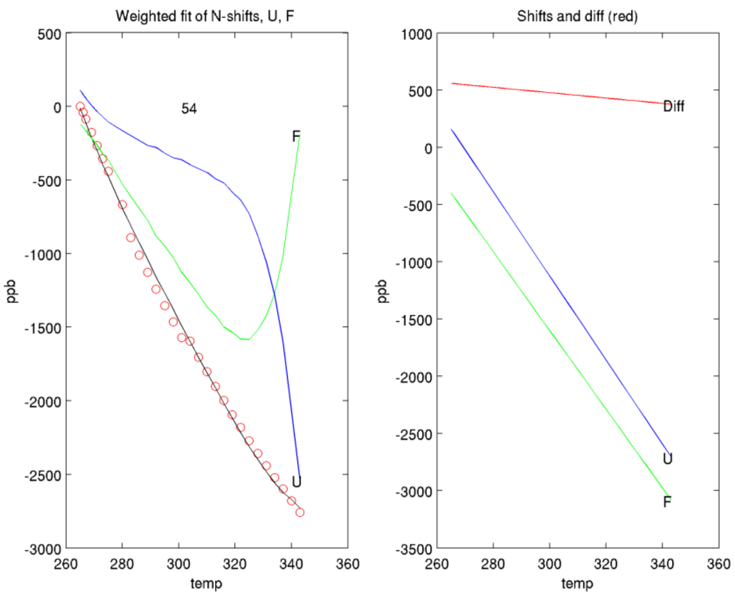

Figure 39: ^{15}N chemical shift fit of Cys-54 based on the populations of folded (F) and unfolded (U) conformers. Left panel with red "o" shows fit (black line) of experimental shifts, right panel displays putative ^{15}N shifts.

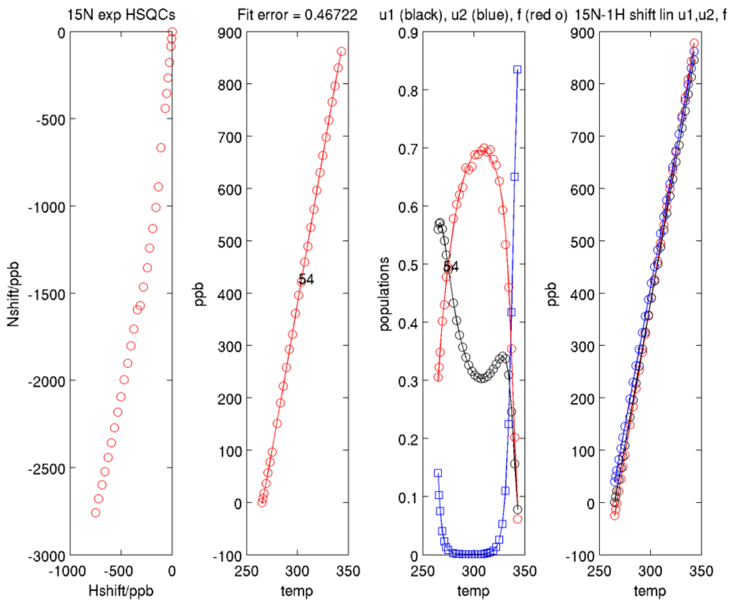

Figure 40: FIU model fit of Cys-54 (panels from left to right: 1. NH-shifts in HSQC spectra, 2. fit of combined NH-shift, 3. populations f, u1 and u2, 4. drift of putative chemical shifts)

Results of ^{15}N-CEST experiments:

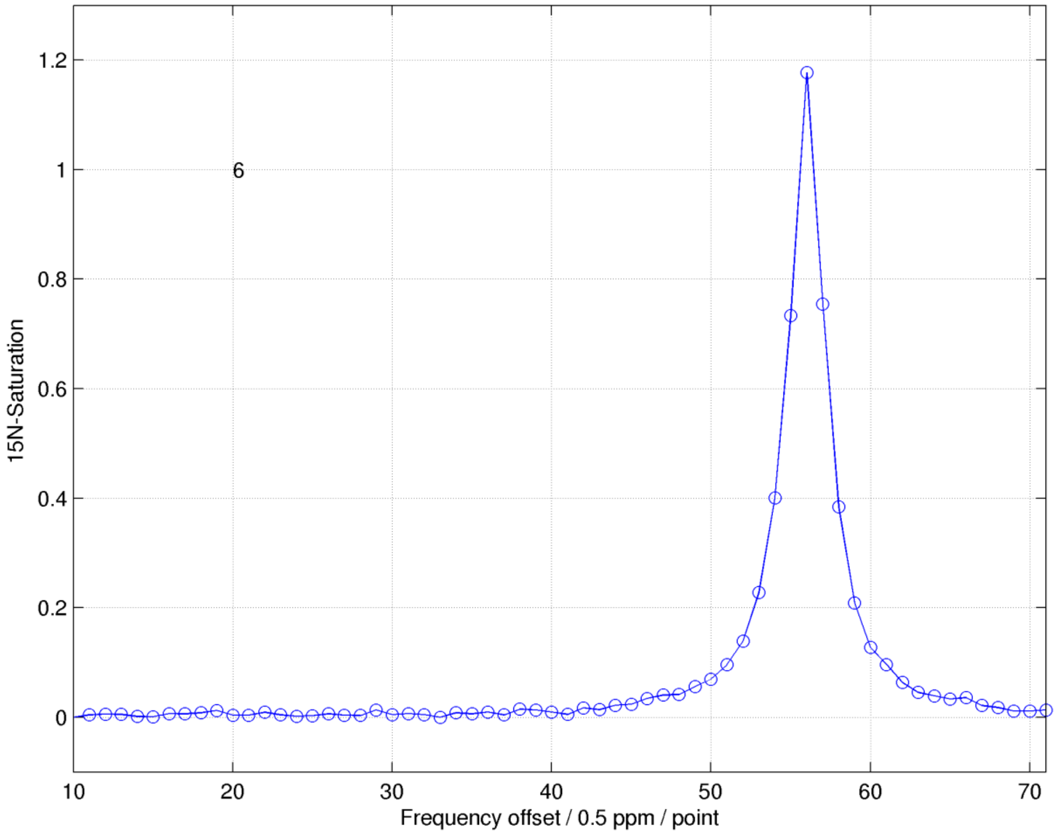

Figure 41. Example of the absence of CEST effect: overlapping signals of Asp23 and Asp26 does not show hidden conformers by CEST-NMR experiment.

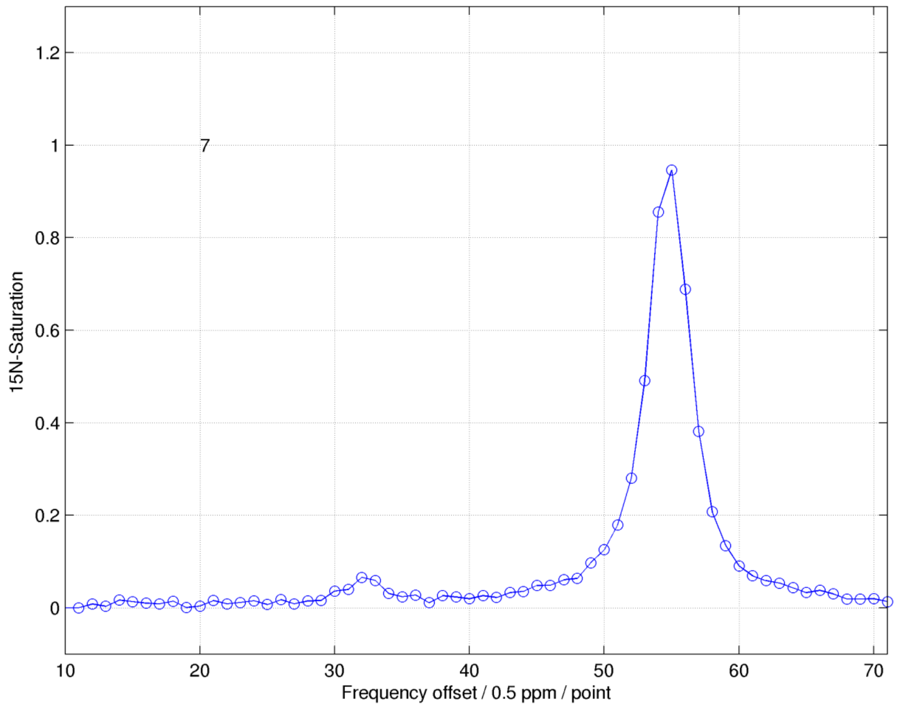

Figure 42: CEST profile of Asp-53 residue at the C-terminal part of PAF

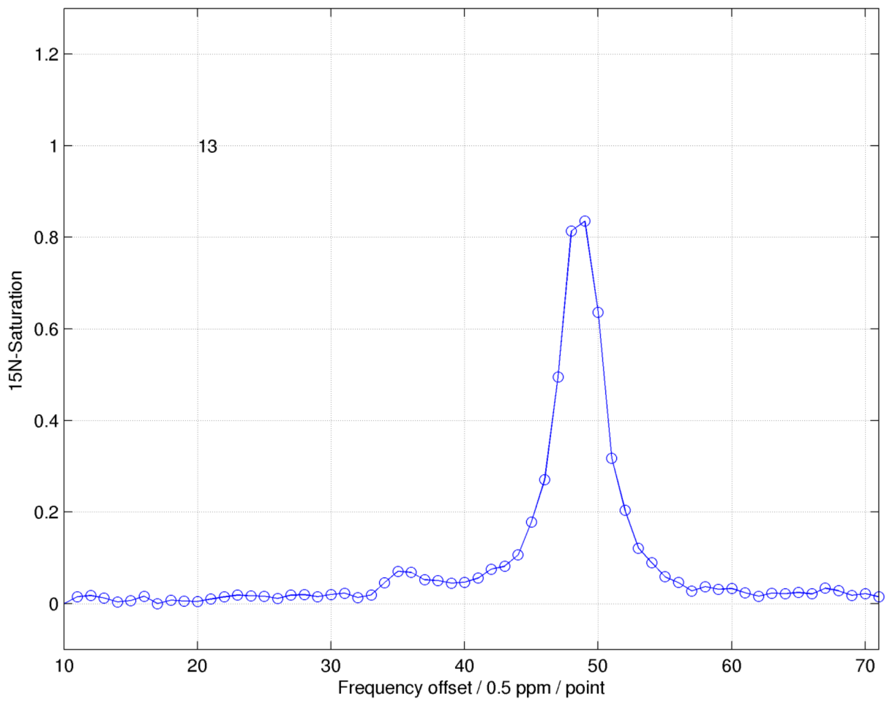

Figure 43: CEST profile of Tyr-3 residue at the N-terminal part of PAF

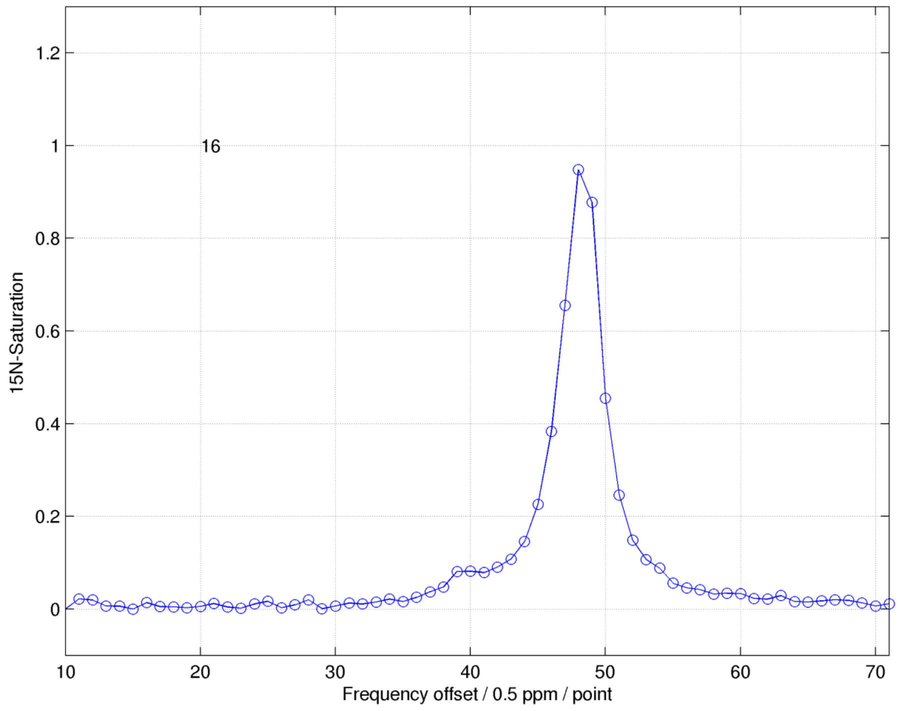

Figure 44: CEST profile of Cys-54 residue at the C-terminal part of PAF

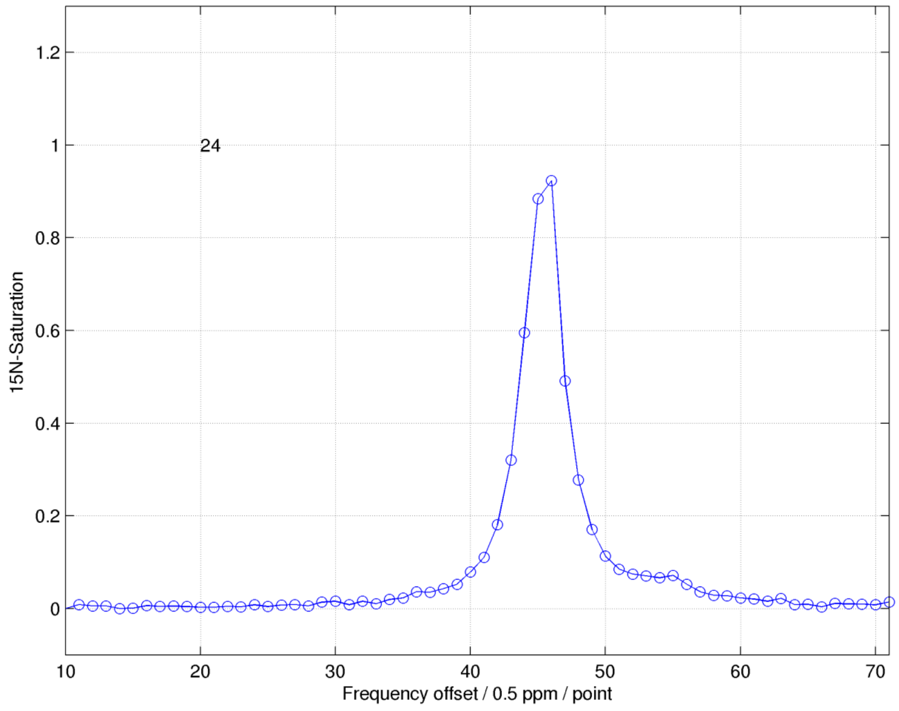

Figure 45: CEST profile of Ala51 residue at the C-terminal part of PAF

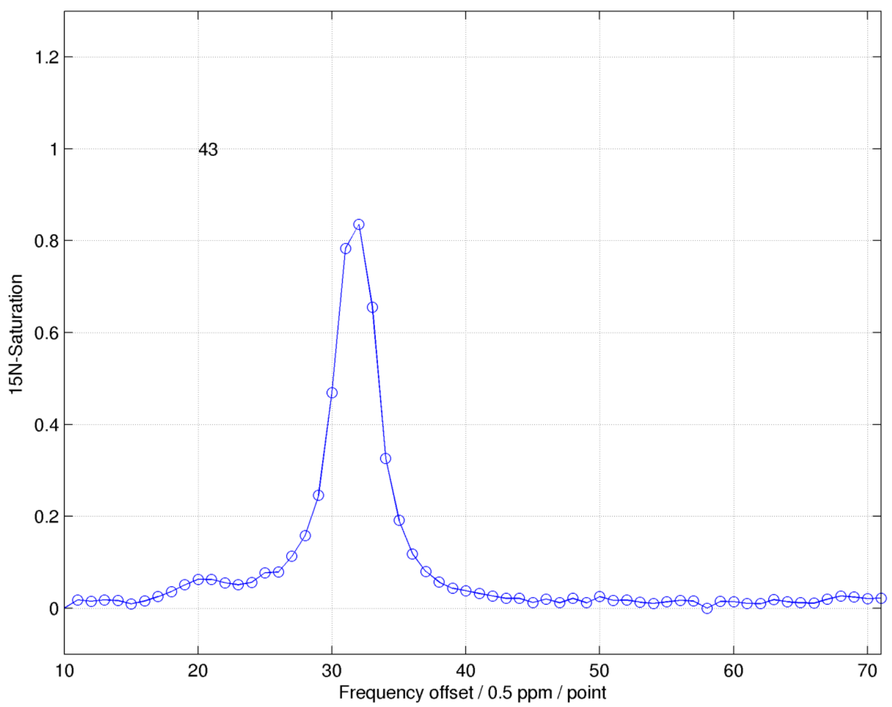

Figure 46: CEST profile of Thr-47 residue at the C-terminal part of PAF.

Results of NMR structure determination and MD simulations

Ensemble name		2MHV	MUMO8	MUMO16
Ensemble size		20	128	256
Backbone RMSD		0.55 ± 0.14	0.94 ± 0.21	0.92 ± 0.22
Heavy RMSD		1.21 ± 0.16	1.51 ± 0.23	1.55 ± 0.23
Rama-chandran statistics	Most favored	92%	86%	86%
	Allowed	8%	13%	13%
	Generously allowed	0%	1%	1%
	Disallowed	0%	0%	0%
N-H S^2	Correlation	0.493	0.904	0.895
	Q-factor	19.70%	3.25%	2.80%
	RMSD	0.16	0.03	0.02
Cα chemical shifts	Correlation	0.958	0.966	0.967
	Q-factor	5.39%	5.59%	5.56%
	RMSD	2.86	2.97	2.96
Hα chemical shifts	Correlation	0.787	0.794	0.801
	Q-factor	7.43%	6.67%	6.62%
	RMSD	0.34	0.31	0.30
N chemical shifts	Correlation	0.740	0.669	0.665
	Q-factor	2.75%	3.12%	3.14%
	RMSD	3.29	3.73	3.76
H-Hα scalar couplings	Correlation	0.628	0.837	0.857
	Q-factor	25.10%	19.16%	18.49%
	RMSD	2.03	1.55	1.50
N-H RDCs	Correlation	0.628	0.718	0.721
	Q-factor	77.69%	70.56%	70.16%
	RMSD	6.24	5.66	5.63

Table 4. Correspondence of structural ensembles to representative experimental data

CEST-detected conformers:

CEST conformers were selected from the conformer pool generated with more aggressive conformational sampling based on their correspondence to observed chemical shifts. The best 4 conformers were selected.

Conformer	Correlation	Q-factor	RMSD
CEST1	0.73	3.03	3.62
CEST2	0.72	3.10	3.72
CEST3	0.73	2.94	3.53
CEST4	0.72	3.04	3.66

Table 5. Correspondence to back-calculated amide N shifts of CEST conformers to observed data (native amide N shifts corrected with CEST-detected offsets for residues 3, 47,51,53 and 54).

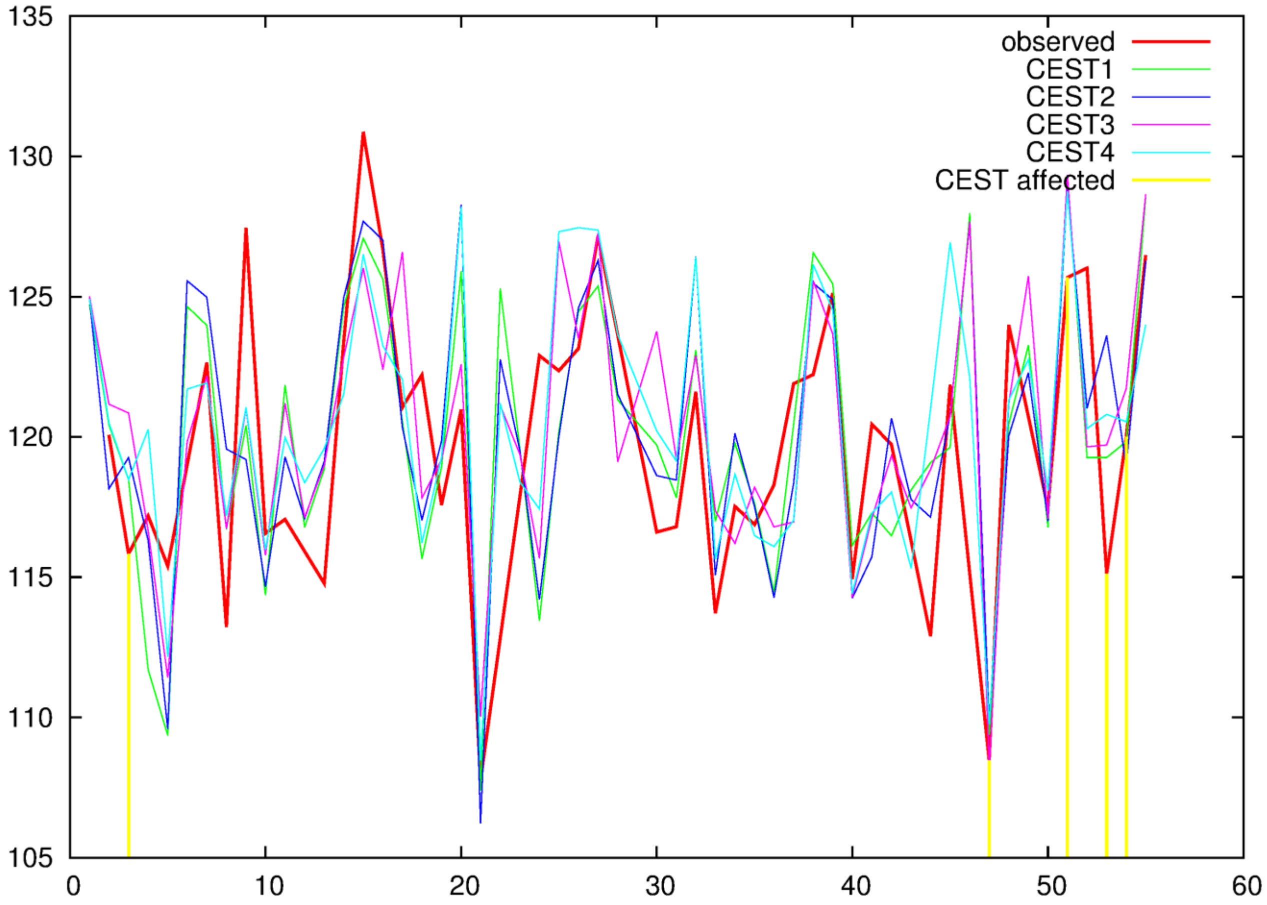

Figure 47: Amide N chemical shifts obtained from CEST experiments and in CEST conformers. Yellow bars denote sites for which CEST effect was observed, thus the chemical shift differs substantially from the native one.

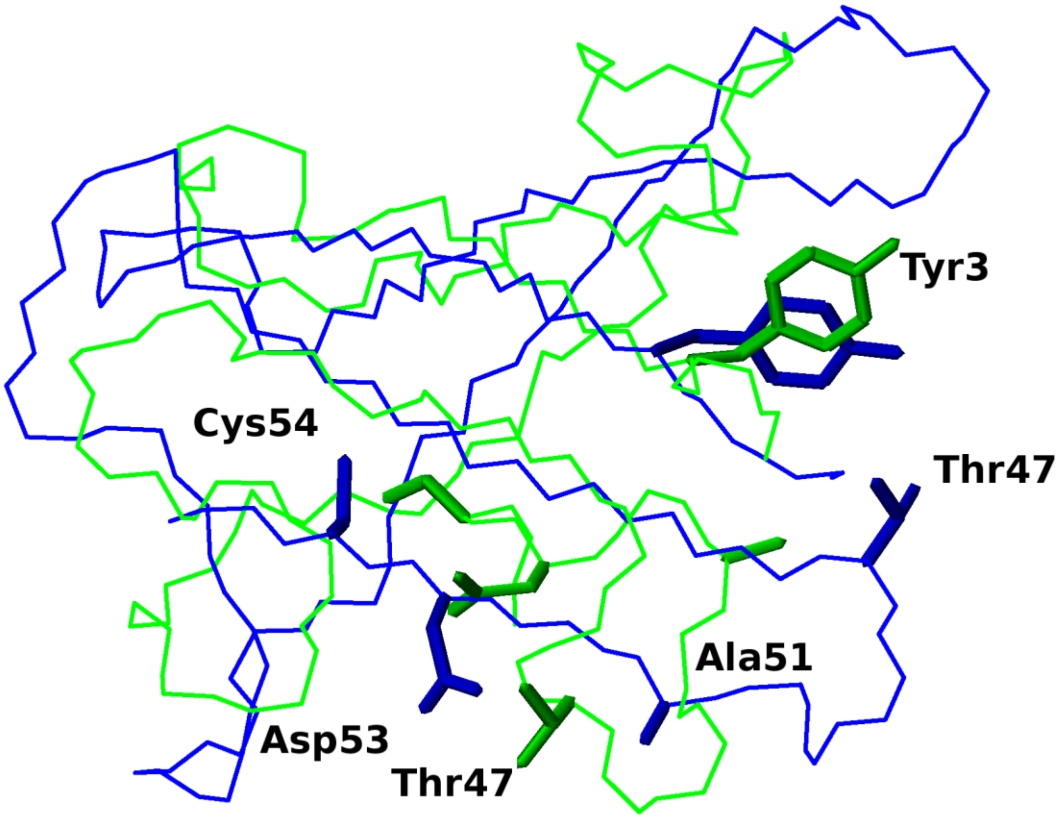

Figure 48: *Red: native blue: the CEST1 conformer. On the left, the disruption of the Tyr3-Thr47 contact in the CEST-detected state can be observed.*

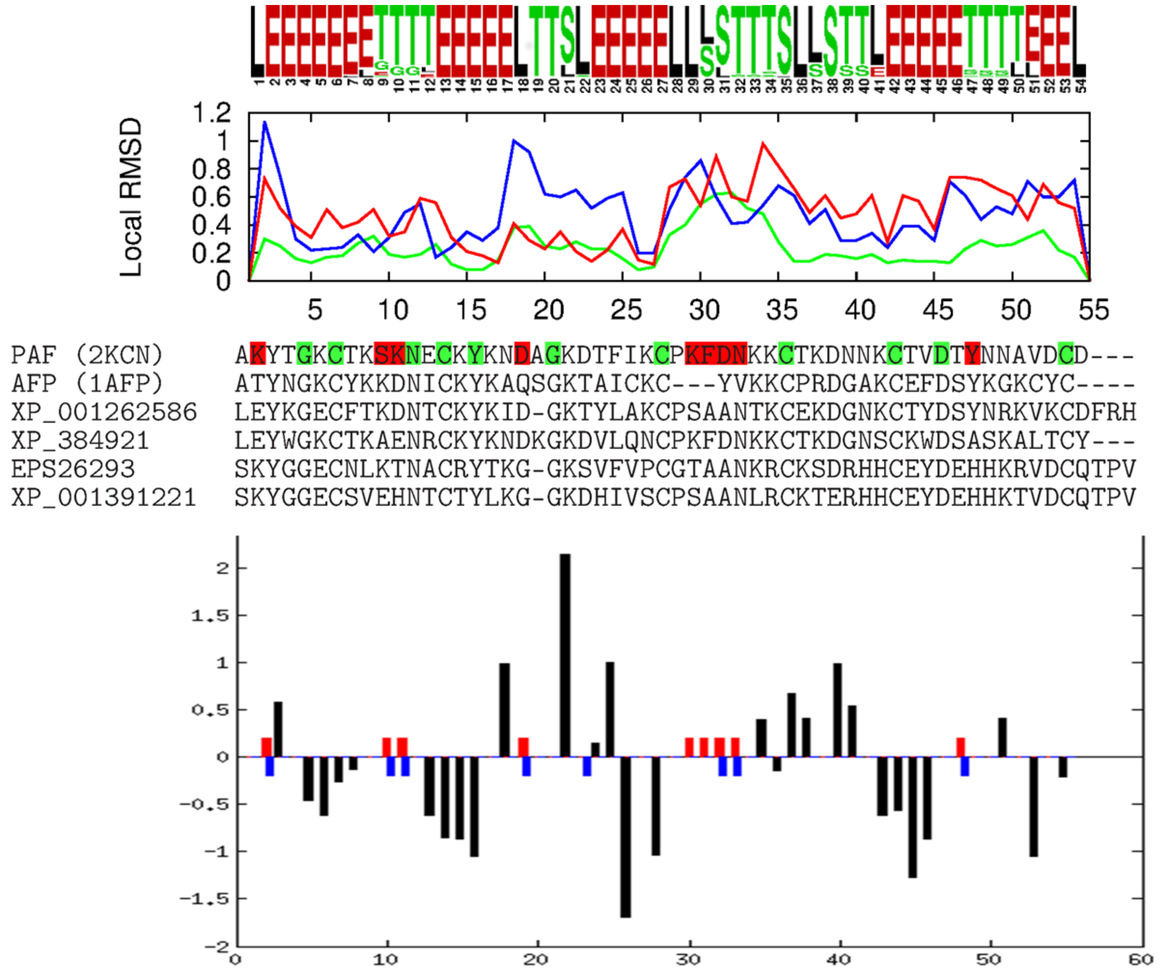

Figure 49.: TOP: secondary structure element in PAF as predicted by DSSPcont. MIDDLE: Local RMSDs between average structures derived from the populations: red line, observable vs. hot state-specific, blue line, observable vs. cold state-specific, green, hot state-specific vs. cold state-specific structures. BOTTOM: sequence alignment of PAF-related proteins. BOTTOM bar diagram: black: amide proton deuteration rates on a log10 scale (1/h). Missing bars are due to very fast NH/ND exchange rates[14]. Red: residues that could be fitted with the two-state model of thermal unfolding, blue: extremely pH sensitive residues.

Temperature (K)	^{15}N T_2 average relaxation times
273	101±7 ms
300	201±32 ms
320	258±25 ms

Table 6. Averaged ^{15}N T_2 values of PAF as a function of temperature. T_2 is a monotonic function of temperature, in agreement with theory.

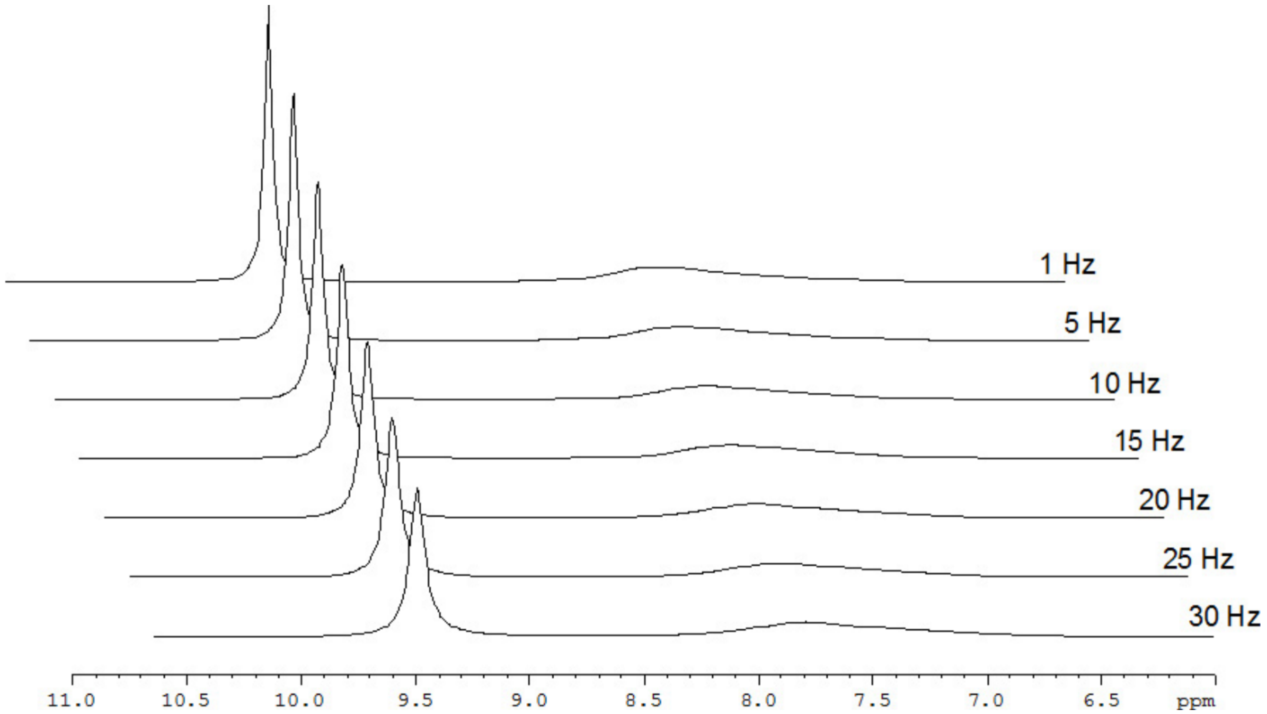

Figure 50.: Three-site exchange simulations using Bruker Topspin 3.0 software that illustrate possible fading effect (loss of integral intensities). 1- x in slow exchange, 2-3 in intermediate exchange. Parameters: LB=20 Hz (line broadening). Chemical shifts and populations are: #1: 9.5 ppm, X1=0.60; #2: 8.0 ppm, X2=0.25; #3: 7.0 ppm, X3=0.15. Exchange rates: 1-2 and 1-3 site rates change parallel as shown by the parameters on the right side of the figure 2↔3 exchange rate is: 850 s^{-1}. Fading effect of the of #3 results fully disappeared signal of the #3 exchange partner. This generates an “invisible” state as “seen” by NMR.

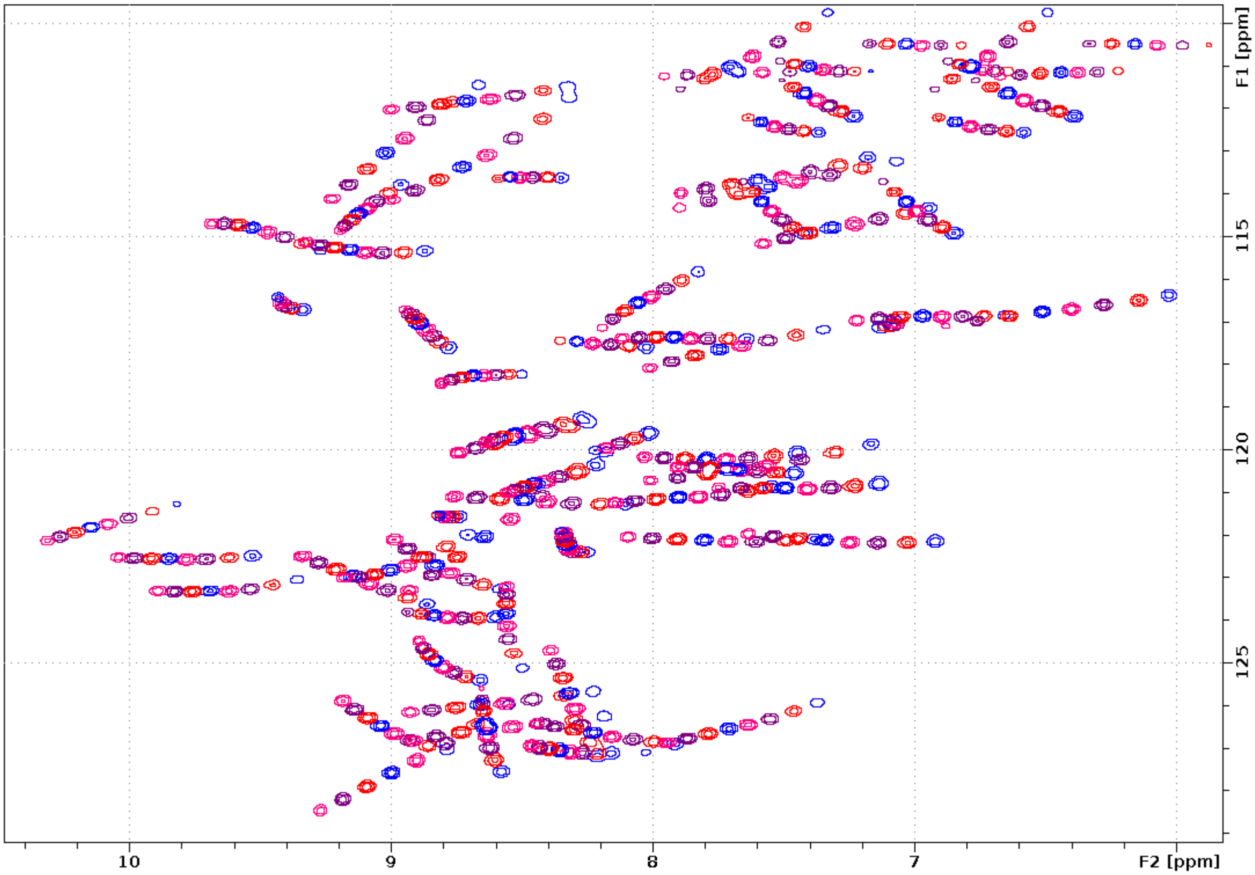

Figure 51. Overlaid ^{15}N-HSQC spectra of PAF roughly in 10K steps from 265K to 344K. The decrease of contour lines is parallel with reduced integral volumes.

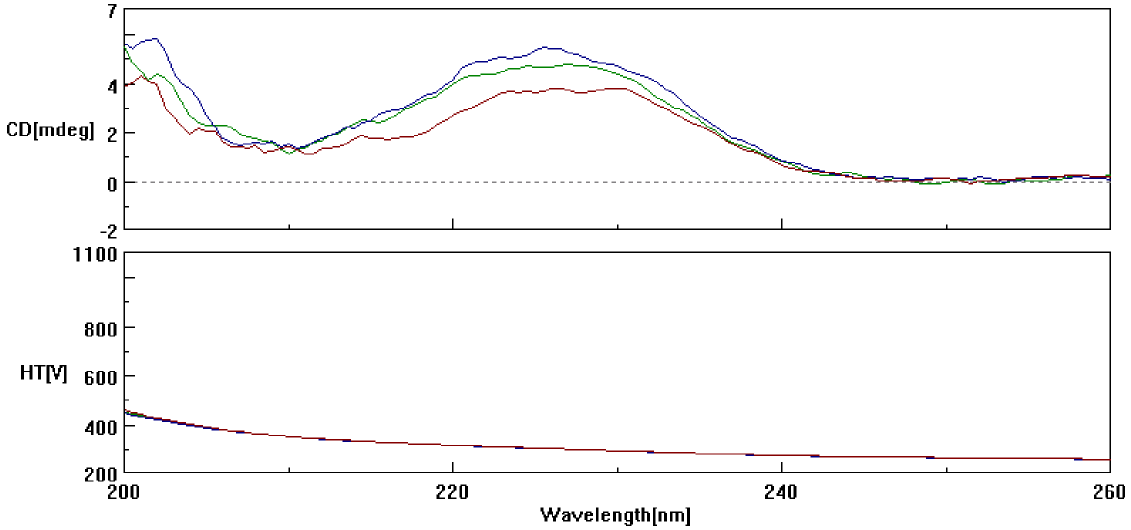

Figure 52. **a.,** ECD spectra of PAF were recorded in a pH 6 phosphate buffer at three temperatures (273K blue, 298K green and 333K brown) using a Jasco-810 electronic circular dichroism spectropolarimeter. The difference between the ECD spectra supports[17] the progress of heat unfolding effect as the temperature is increasing. ECD effect arises mostly from disulfide bonds buried in the hydrophobic core, while NMR is most sensitive to the exterior of PAF due to observing the NH amide signals.

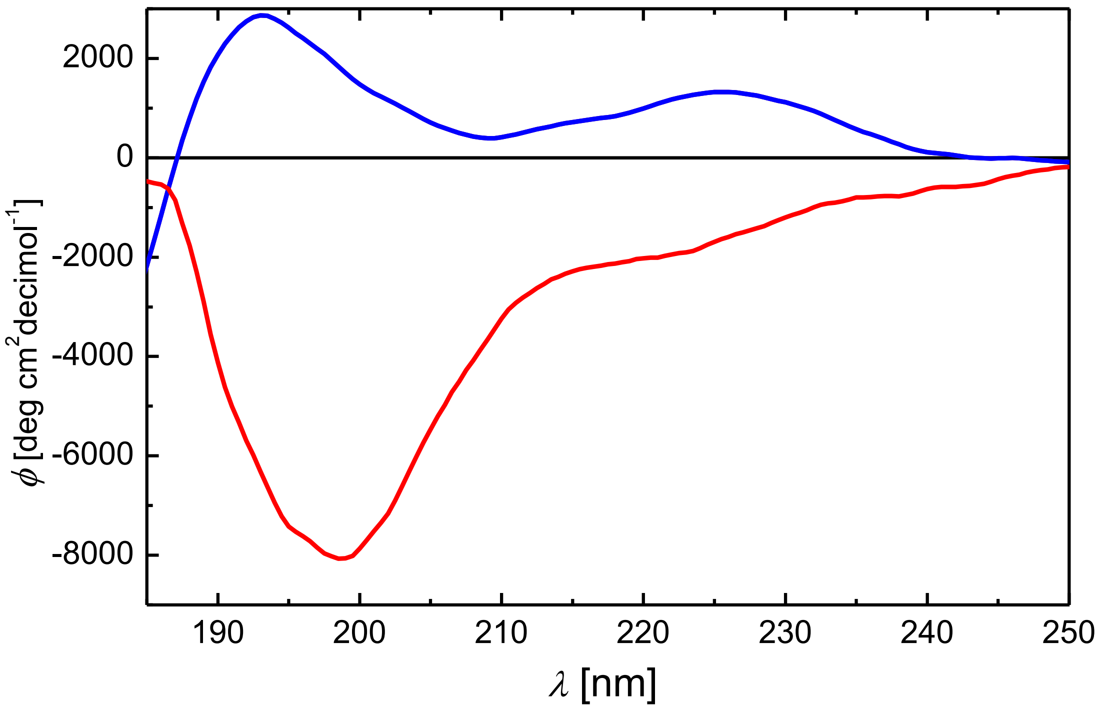

Figure 52. **b.,** ECD spectra of PAF (blue curve, wild type) and synthetic, linear PAF [18] (red curve). The wild-type PAF does not show the characteristic ECD features of its dominant β-sheet secondary structure because of the presence of disulfide bonds. The positive Cotton effects (CE) of the wild-type PAF at 226

and 193 nm are most likely governed by the n-σ* transitions of the inherently chiral disulfide chromphores, and are mostly affected by the dihedral angles of the disulfide bonds. In the linear PAF, negative CE appear, in agreement with its unfolded structure.

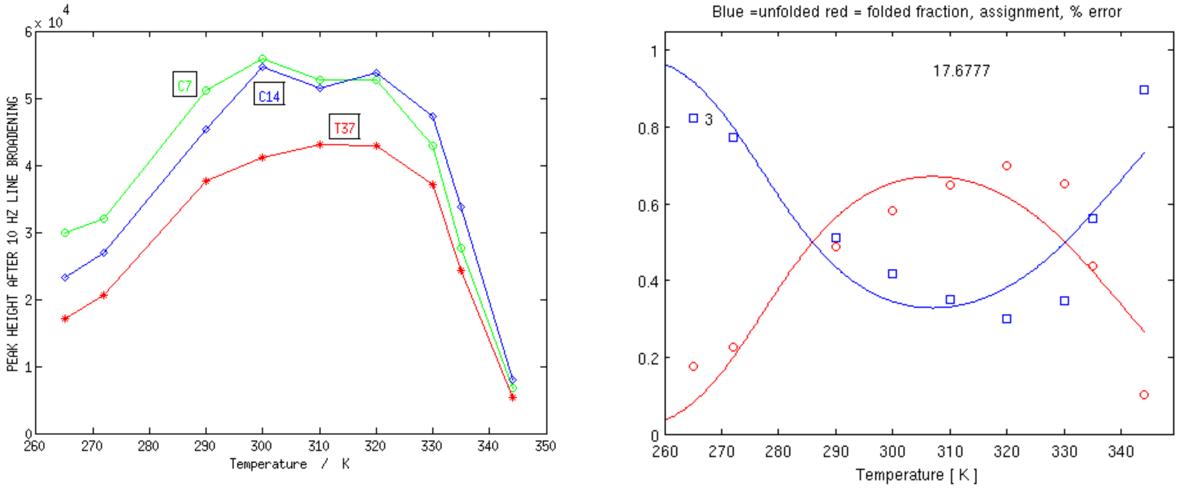

Figure 53a(left), 53b(right): Impact of strong exponential weighting function (line broadening, LB =10 Hz) on the evaluation of ^{15}N-HSQC peak volume integrals.

We reprocessed nine representative ^{15}N-HSQC experiments and chose three well separated HSQC peaks (C7, C14, T37). Instead of straight volume integration of cross-peaks, the amplitudes of the artificially broadened peaks were considered to be proportional with the peak volumes('PULCON' corrections have been considered). This procedure has been applied to peaks C7, C14 and T37. Undoubtedly, this alternative "peak amplitude" method shows population maxima at intermediate temperatures, and gives T (max_stability) = 307 ± 2K. The obtained average thermodynamic parameters for the three residues are in good agreement with the average of group-2 residues (Supplementary Table 1.) : ΔH_u=52 ± 2 kJ/M , T_h = 330 ± 2K, T_l =284 ±3K, ΔC_p = 2.2 ± 0.1 kJ/M/K , $\Delta G_{u,max}$ = 1.9± 0.1 kJ/M/. However, the fitting errors are somewhat bigger if compared to straight volume integrals. As a conclusion we can say that an alternative processing method, free of T_2 and volume integration caveats, yielded similar results to the straight peak volume integral method, though at the expense of somewhat larger fit errors. Figure 53a shows the raw data, while Fig 53b shows the result of two-state fitting of unfolding for T37. Consequently, T_2 effects are unlikely to influence the results obtained by the ^{15}N-HSQC volume integration method for a small protein like PAF.

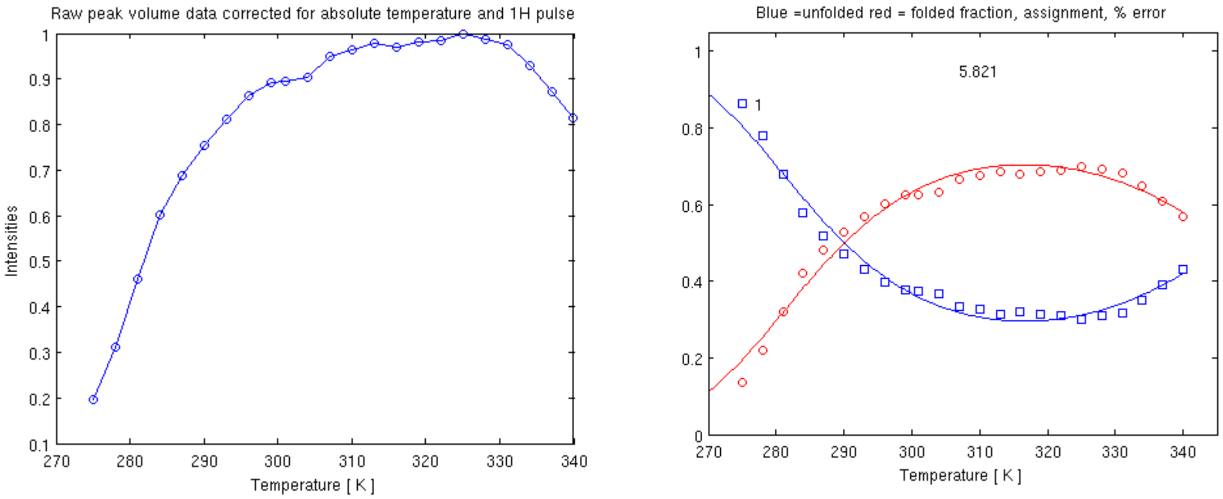

Figure 54a(left), 54b(right): Results of 1D-1H NMR single peak method and comparison with the results obtained from 2D ^{15}N-HSQC spectra.

A series of 1D ^{1}H-NMR spectra of PAF in D_2O/phosphate buffer has been recorded as a function of temperature. Even for this small protein it is difficult to find lone peaks that can be monitored in a broad temperature range without overlap. We monitored the aromatic $H\varepsilon$ proton of Tyr-16, corrected the integrals according to 'PULCON' and fitted it using the Becktel-Schellman two-state model. In order to reduce possible T_2 effects on peak areas, strong exponential weighting function was applied on the FIDs before Fourier transformation, using the line broadening parameter LB=10. The peak areas were integrated after careful baseline correction. Fig. 54a shows the raw 1D peak area data, while Fig. 54b displays the results of two-state fitting yielding thermodynamic parameters as ΔH_u = 55 kJ/M , T_h = 346K, T_l = 290K , ΔC_p = 1.8 kJ/M/K , $\Delta G_{u,max}$ = 2.3 kJ/M. The parameters of thermal unfolding of the Tyr-16 residue obtained by the 1D 1H-NMR method are in reasonable agreement with the average of group-2 residues (Table 1).

Supplementary References:

(1) Becktel, W. J.; Schellman, J. A. *Biopolymers* **1987**, *26*, 1859.

(2) Privalov, P. L.; Gill, S. J. *Advances in Protein Chemistry* **1988**, *39*, 191.

(3) Privalov, P. L. *Critical Reviews in Biochemistry and Molecular Biology* **1990**, *25*, 281.

(4) Szyperski, T.; Mills, J. L.; Perl, D.; Balbach, J. *European Biophysics Journal with Biophysics Letters* **2006**, *35*, 363.

(5) Kumar, S.; Tsai, C. J.; Nussinov, R. *Biochemistry* **2002**, *41*, 5359.

(6) Pastore, A.; Martin, S. R.; Politou, A.; Kondapalli, K. C.; Stemmler, T.; Temussi, P. A. *Journal of the American Chemical Society* **2007**, *129*, 5374.

(7) Best, R. B.; Vendruscolo, M. *Journal of the American Chemical Society* **2004**, *126*, 8090.

(8) Richter, B.; Gsponer, J.; Varnai, P.; Salvatella, X.; Vendruscolo, M. *Journal of Biomolecular Nmr* **2007**, *37*, 117.

(9) Angyan, A. F.; Szappanos, B.; Perczel, A.; Gaspari, Z. *Bmc Structural Biology* **2010**, *10*.

(10) Hamelberg, D.; Mongan, J.; McCammon, J. A. *Journal of Chemical Physics* **2004**, *120*, 11919.

(11) Onufriev, A.; Case, D. A.; Bashford, D. *Journal of Computational Chemistry* **2002**, *23*, 1297.

(12) Han, B.; Liu, Y. F.; Ginzinger, S. W.; Wishart, D. S. *Journal of Biomolecular Nmr* **2011**, *50*, 43.

(13) Bakan, A.; Meireles, L. M.; Bahar, I. *Bioinformatics* **2011**, *27*, 1575.

(14) Batta, G.; Barna, T.; Gaspari, Z.; Sandor, S.; Kover, K. E.; Binder, U.; Sarg, B.; Kaiserer, L.; Chhillar, A. K.; Eigentler, A.; Leiter, E.; Hegedus, N.; Pocsi, I.; Lindner, H.; Marx, F. *Febs Journal* **2009**, *276*, 2875.

(15) Lipari, G.; Szabo, A. *Journal of the American Chemical Society* **1982**, *104*, 4546.

(16) Kroenke, C. D.; Loria, J. P.; Lee, L. K.; Rance, M.; Palmer, A. G. *Journal of the American Chemical Society* **1998**, *120*, 7905.

(17) L. Whitmore, B. A. Wallace, *Biopolymers* **2008**, *89*, 392–400.

(18) G. Váradi, G. K. Tóth, Z. Kele, L. Galgóczy, Á. Fizil, G. Batta*, Chem. - A Eur. J.* **2013**, 19, 12684–12692.
